# An optimized growth medium for increased recombinant protein secretion titer via the type III secretion system

**DOI:** 10.1186/s12934-021-01536-z

**Published:** 2021-02-15

**Authors:** Lisa Ann Burdette, Han Teng Wong, Danielle Tullman-Ercek

**Affiliations:** 1 Department of Chemical and Biomolecular Engineering, University of California-Berkeley, Berkeley, USA; 2 Present Address: Department of Chemical and Biological Engineering, Northwestern University, Evanston, IL 60208 USA; 3 Department of Plant and Microbial Biology, University of California-Berkeley, Berkeley, USA; 4 Present Address: Institute of Molecular and Cell Biology, 61 Biopolis Way, Singapore, 138673 Singapore

**Keywords:** Protein secretion, T3SS, Recombinant protein, Salmonella, Media optimization

## Abstract

**Background:**

Protein secretion in bacteria is an attractive strategy for heterologous protein production because it retains the high titers and tractability of bacterial hosts while simplifying downstream processing. Traditional intracellular production strategies require cell lysis and separation of the protein product from the chemically similar cellular contents, often a multi-step process that can include an expensive refolding step. The type III secretion system of *Salmonella enterica* Typhimurium transports proteins from the cytoplasm to the extracellular environment in a single step and is thus a promising solution for protein secretion in bacteria. Product titer is sensitive to extracellular environmental conditions, however, and T3SS regulation is integrated with essential cellular functions. Instead of attempting to untangle a complex web of regulatory input, we took an “outside-in” approach to elucidate the effect of growth medium components on secretion titer.

**Results:**

We dissected the individual and combined effects of carbon sources, buffers, and salts in a rich nutrient base on secretion titer. Carbon sources alone decreased secretion titer, secretion titer increased with salt concentration, and the combination of a carbon source, buffer, and high salt concentration had a synergistic effect on secretion titer. Transcriptional activity measured by flow cytometry showed that medium composition affected secretion system activity, and prolonged secretion system activation correlated strongly with increased secretion titer. We found that an optimal combination of glycerol, phosphate, and sodium chloride provided at least a fourfold increase in secretion titer for a variety of proteins. Further, the increase in secretion titer provided by the optimized medium was additive with strain enhancements.

**Conclusions:**

We leveraged the sensitivity of the type III secretion system to the extracellular environment to increase heterologous protein secretion titer. Our results suggest that maximizing secretion titer via the type III secretion system is not as simple as maximizing secreted protein expression—one must also optimize secretion system activity. This work advances the type III secretion system as a platform for heterologous protein secretion in bacteria and will form a basis for future engineering efforts.

## Background

The ever-increasing diversity of commercial protein products coupled with the high cost of developing these products is driving engineering efforts to develop low-cost, easy-to-manipulate production systems. Bacterial hosts are robust, genetically tractable, and inexpensive to cultivate, but traditional intracellular expression strategies present several challenges. Products must be recovered from a complex lysate mixture, which requires several downstream purification steps [1, 2]. Further, intracellular overexpression of heterologous proteins often causes aggregation in insoluble inclusion bodies. Initial product purity is often higher in inclusion bodies, but resolubilization and refolding processes must be developed for each product [3]. Finally, many heterologous proteins, including biomaterials such as spider silk, are difficult to express at high levels because they are toxic to the host [4, 5]. Engineering bacteria to secrete heterologous proteins into the extracellular space eliminates these constraints.

The type III secretion system (T3SS) is a multimeric protein needle complex that spans the inner and outer membranes of the bacterial cell and secretes proteins from the cytoplasm to the extracellular space in a single step [6]. The T3SS is not required for cell viability, which facilitates engineering efforts and allows it to be used solely for secreting heterologous proteins. The *Salmonella* pathogenicity island 1 (SPI-1) T3SS of *Salmonella enterica* Typhimurium has been successfully engineered to secrete high titers of several heterologous proteins, including spider silk monomers, antimicrobial peptides, and single chain variable fragments (scFvs) [4, 7, 8].

Using the SPI-1 T3SS for recombinant protein production is complicated by its sensitivity to environmental input. Traditionally, secretion is activated by shifting cells to high osmolarity, low aeration growth conditions [7, 9]. These conditions yield low secretion titers because maximum achievable cell density is low and only ~ 33% of cells have T3SS activity. In prior work, we showed that overexpressing the SPI-1 T3SS master regulator HilA relieved these restrictions and increased secretion titer by an order of magnitude [10]. HilA overexpression activated the T3SS in more than 90% of the population and facilitated secretion in low-salt medium with moderate aeration, allowing higher cell densities. In subsequent work, combining HilA overexpression, moderate aeration, and a high osmolarity medium increased secretion titer further [8].

Despite these successes, secretion titers remain below 1 g/L, which makes it difficult to compete with existing production technologies that achieve titers at or above 1 g/L [11–14]. Moreover, the T3SS responds to numerous extracellular inputs in addition to osmolarity and oxygen, including pH and nutrient concentrations [15–17]. This sensitivity is difficult to circumvent or engineer directly because the SPI-1 regulatory network is complex—it receives input from regulatory systems that are essential for normal cellular function such as the DNA-binding protein HN-S, the Csr carbon storage regulatory system, and the osmolarity sensor OmpR/EnvZ [18–20].

Given this complexity, in this work we reasoned that instead of attempting to relieve T3SS repression at the genetic level, we could take an “outside-in” approach and engineer growth medium composition to identify and optimize environmental factors that promote consistently high secretion titers. We screened common rich bacterial growth media and found that carbon sources, buffering agents, and ionic content were critical factors for maximizing T3SS secretion titer. Individually, non-ionic carbon sources repressed secretion via the T3SS while high ionic content increased secretion titers. Adding a buffering agent prevented the decrease in secretion titer observed in the presence of non-ionic carbon sources, and the combination of a non-ionic carbon source, a buffering agent, and high ionic content had a synergistic effect on secretion titer. SPI-1 T3SS transcriptional activity showed that this optimal combination increased secretion titer in part by elevating transcriptional activity and prolonging secretion system activation. An optimized combination of glycerol, potassium phosphate, and sodium chloride increased secretion titer at least fourfold for several model proteins and was additive with strain improvements.

## Results

### Growth medium affects secretion titer

An optimal medium for protein production is composed of nutrients that maximize cell density and protein production per cell, or specific productivity. In our prior work, we found that overexpressing the master regulator HilA increased bulk secretion titer by activating secretion in over 90% of the cell population in conditions that maximized cell density [10]. We observed an increase in specific productivity, however, only when we overexpressed HilA in LB-IM media (Lysogeny Broth containing 17 g/L NaCl) [8]. This finding highlights a unique challenge for a protein production platform that makes use of the T3SS—secretion titer is increased in high-salt conditions that limit cell density. As a result, an ideal growth medium for protein production via the T3SS will need to strike the optimal balance between maximizing cell density and specific productivity.

Though numerous environmental inputs to the T3SS are documented [15–17], they were not characterized in the context of our engineered system. Thus, to expand our knowledge of medium components that affect secretion titer, we began by simply measuring expression and secretion titer of a model protein in the common bacterial growth media 2X YT and terrific broth (TB). The model protein, or protein of interest (POI), was the soluble catalytic DH domain from the human protein intersectin-1L fused C-terminal to the T3SS secretion tag SptP to facilitate secretion [7, 21]. SptP-DH-2xFLAG-6xHis was expressed from an “export vector” under the control of the native SPI-1 promoter P_*sic*_. It was co-transformed with a “secretion activation vector” containing a cassette for inducible HilA overexpression into *S. enterica* Typhimurium ASTE13. This two-plasmid system enabled T3SS activation and POI expression with a single induction event [7, 10]. SptP-DH secretion titers were measured relative to LB-L using semi-quantitative western blotting.

**Fig. 1:**
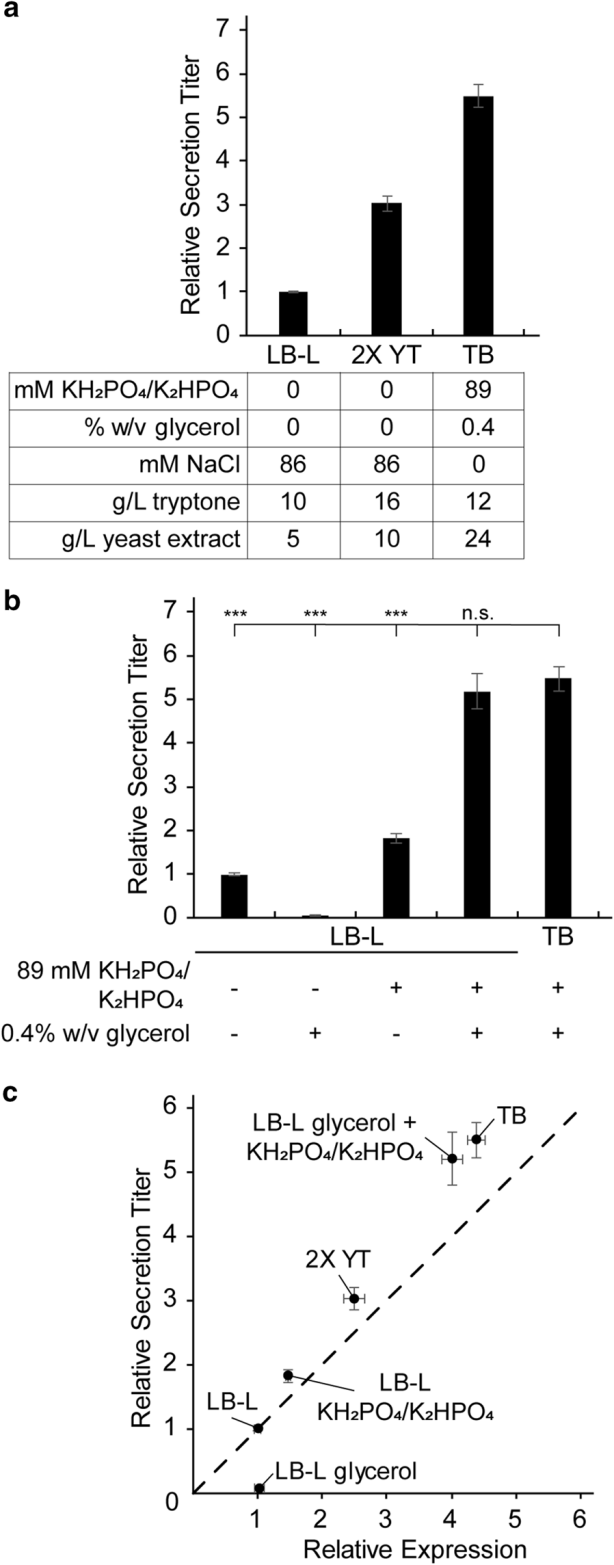
Medium composition affects secretion titer. **a** Relative bulk secretion titer of SptP-DH-2xFLAG-6xHis in LB-L, 2X YT, and TB. **b** Relative bulk secretion titer of SptP-DH-2xFLAG-6xHis in LB-L supplemented with the defined components in TB. Statistical significance was determined by Welch’s unequal variances t-test. Significance denoted by “***” represents p < 0.001, while “n.s.” represents p > 0.05. **c** Relative bulk secretion titer versus relative expression in the media from parts **a**, **b**. Expression and secretion titer were normalized to LB-L with no carbon source using densitometry measurements on western blots. All error bars represent standard error for four biological replicates

Bulk secretion titer increased threefold in 2X YT and 5.5-fold in TB relative to LB-L (Fig. 1a). 2X YT and TB increased expression of SptP-DH 2.5-fold and 4.5-fold (Fig. 1c, Additional file 1: Figure S1) suggesting that an increase in secretion titer correlates with an increase in expression of the target protein. The increase in secretion titer closely matched the increase in expression in 2X YT, but the relative increase in secretion titer was 20% higher than the relative increase in SptP-DH expression in TB (Fig. 1c). This discrepancy suggested that some components in TB caused a higher percentage of expressed protein to be secreted. We will henceforth refer to the fraction of expressed protein that is secreted as “secretion efficiency”.

TB contains two defined components that are not present in LB-L or 2X YT: 0.4% w/v glycerol and 89 mM potassium phosphate (Fig. 1a). We hypothesized that adding those components to LB-L could recapitulate the effects observed in TB. Surprisingly, separate addition of those components to LB-L revealed a competing dynamic—glycerol repressed secretion, while phosphate buffer promoted secretion. Combining the two components in LB-L, however, had a synergistic effect, matching the increase in secretion titer and efficiency observed in TB (p < 0.001 for separate addition, p = 0.6 for LB-L with glycerol and phosphate)(Fig. 1b, c).

### Non-ionic carbon sources decrease secretion titer

SPI-1 T3SS transcriptional activity is repressed in the presence of glucose [22], so the discovery that glycerol negatively impacted secretion titer led us to screen a panel of carbon sources in LB-L. Our goal was to determine the optimal carbon source for maximum secretion titer, as well as to develop an understanding of how these carbon sources might be affecting secretion titer through heterologous protein expression and T3SS expression. Therefore, we evaluated SptP-DH secretion titer, expression, and T3SS transcriptional activity for each carbon source in LB-L. Secretion titer and expression were measured relative to LB-L using semi-quantitative western blotting. Transcriptional activity over time was measured by performing flow cytometry on strains with GFP integrated into one of the SPI-1 *inv*, *prg*, or *sic* loci. The *inv*, *prg*, and *sic* operons encode regulatory, structural, and natively secreted proteins, respectively [23].

**Fig. 2:**
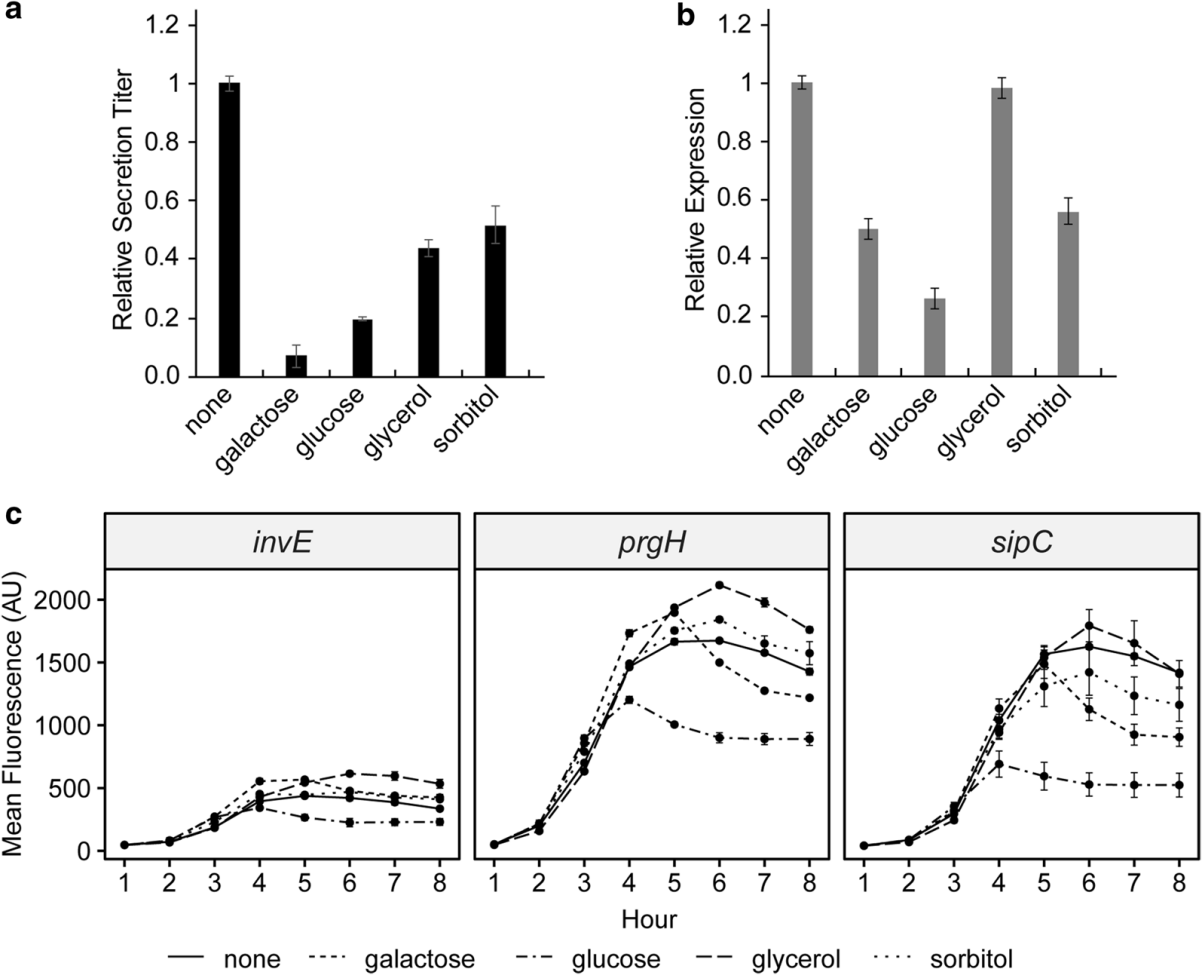
Carbon sources decrease secretion titer in LB-L. **a** Relative bulk secretion titer and **b** expression of SptP-DH-2xFLAG-6xHis. Expression and secretion titer were normalized to LB-L with no carbon source using densitometry measurements on western blots. **b** Transcriptional activity at the *inv*, *prg*, and *sic* loci was measured by performing flow cytometry on strains with transcriptional fusions of GFPmut2 at the appropriate locus. All error bars represent standard error for three biological replicates

SptP-DH secretion titer decreased relative to no added carbon source in the presence of all carbon sources tested (Fig. 2a), and SptP-DH expression decreased in the presence of all carbon sources except glycerol (Fig. 2b). Cell density was approximately constant across all conditions (Mean OD_600nm_ ± S.D., Additional file 1: Table S5), indicating that the added carbon sources altered SptP-DH expression and secretion titer by affecting T3SS function. T3SS transcriptional activity, as assessed by flow cytometry, supported this reasoning (Fig. 2c, Additional file 1: Figure S4). Glucose had a strong negative effect on expression and secretion titer and caused lower transcriptional activity that ceased earlier than all other carbon sources. Glycerol, conversely, caused the smallest decrease in secretion titer (statistically equivalent to sorbitol, p = 0.34), had no effect on expression relative to no added carbon source, and transcriptional activity in that condition was higher than and showed similar dynamics to no added carbon source.

As a visual examination of the flow cytometry data suggests, the maximum transcriptional activity, represented by maximum fluorescence, and the length of transcriptional activation correlated with SptP-DH expression and secretion titer (Additional file 1: Figure S4A-B). Flow cytometry measures transcriptional activity on a per cell basis, so its results were compared to SptP-DH expression and secretion titer per cell. Spearman correlations revealed that SptP-DH expression per cell correlated with both the level of transcriptional activation at T3SS loci (Additional file 1: Figure S4B), and the duration of T3SS activation (Additional file 1: Figure S4A). SptP-DH secretion titer per cell correlated only with the duration of T3SS activation. The strength of the correlations varied by locus and parameter—*invE* activity was least predictive for either expression or secretion titer, while maximum activation and length of activation at *sipC* was most predictive. Taken together, these results suggest that prolonged T3SS activity is a critical factor in maximizing secretion titer, but that alone does not explain the observed decreases in secretion titer. That effect might also be explained by acidification of the medium (Additional file 1: Table S5), an environmental condition known to repress secretion via the SPI-1 T3SS [16, 17].

### Secretion titer increases with ionic content

Phosphate is a unique buffer species because in addition to providing buffer capacity, it contributes significantly to the ionic content of the medium. To decouple the effects of buffering and increased ionic content, we compared secretion titer in media containing a base of 10 g/L tryptone and 5 g/L yeast extract supplemented with a range of concentrations of potassium phosphate, 3-(*N*-morpholino)propanesulfonic acid (MOPS), sodium chloride, or MOPS supplemented with sodium chloride (Additional file 1: Table S6). We selected MOPS as an alternative buffer species because it is the main buffer component in a defined medium explicitly designed for *S. enterica* cultivation [24], it has a minimal contribution to ionic strength, and it is one of Good’s buffers [25]. To mimic the simultaneous contributions of buffering and ionic content inherent to phosphate, we supplemented MOPS with sodium chloride. Finally, to control for changes in ionic content in the absence of a buffering agent, we tested sodium chloride alone. We monitored ionic content by measuring conductivity. The concentrations of sodium chloride with and without MOPS were chosen to match the conductivities of the specified concentrations of potassium phosphate. SptP-DH expression and secretion titer were compared to that in LB-L with no additives using semi-quantitative western blotting.

**Fig. 3:**
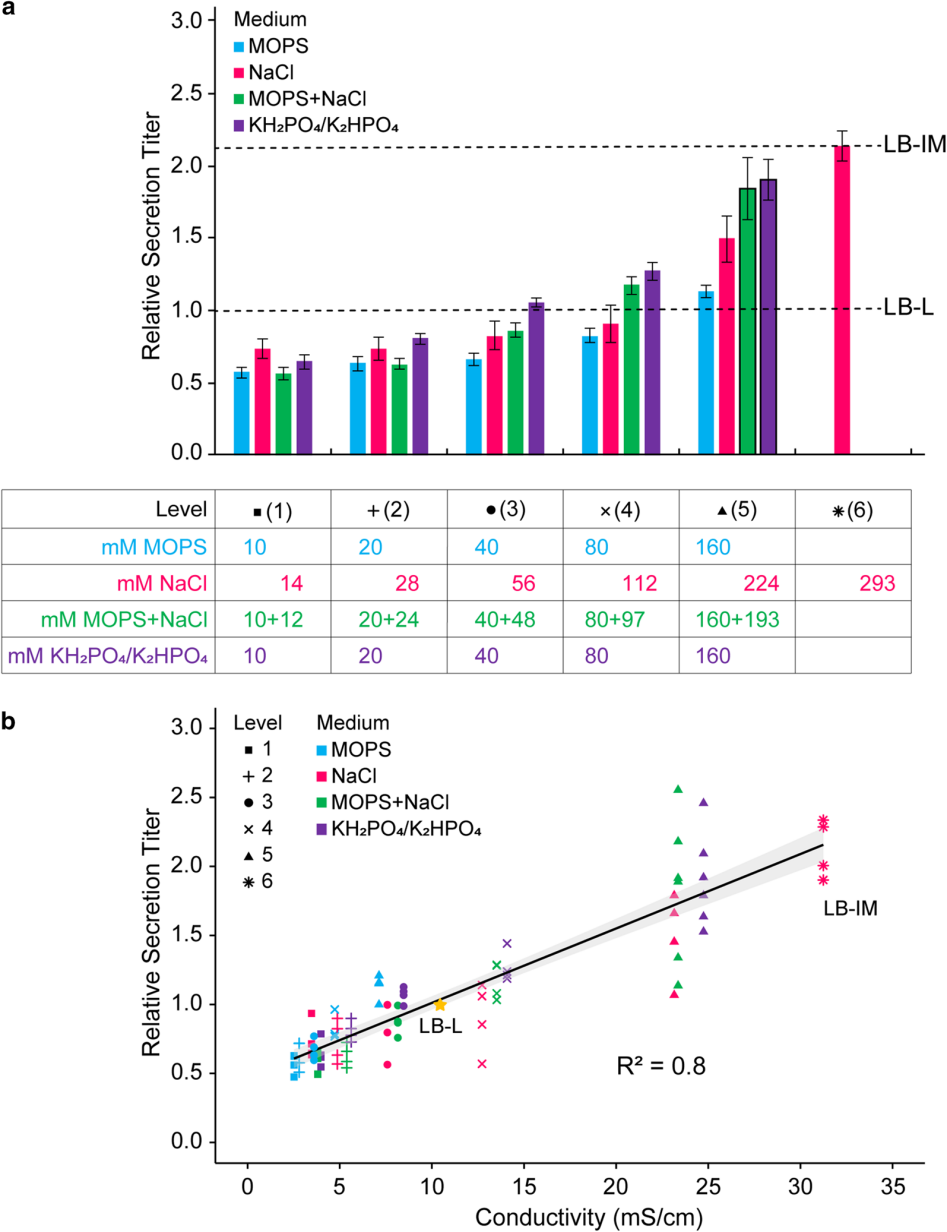
Secretion titer increases with conductivity irrespective of species. **a** Relative bulk secretion titer of SptP-DH-2xFLAG-6xHis in LB supplemented with the buffers and salts listed below the graph. Species in each “level” were designed to have a similar buffer concentration (MOPS, MOPS + NaCl) and/or conductivity (NaCl, MOPS + NaCl) to the corresponding concentration of KH_2_PO_4_/K_2_HPO_4_. Each column of the table corresponds to the group of bars above it, and each row of the column corresponds to a separate bar in the group. Data was normalized to LB-L using semi-quantitative western blotting. Error bars represent standard error for four biological replicates except for black-outlined bars, which represent six biological replicates. LB-L and LB-IM are denoted by dashed lines. **b** Relative bulk secretion titer versus conductivity for the media in part **a**. The gray shading represents a 95% CI on the linear regression. Each data point is a replicate except for LB-L, which is labeled as a reference

Expression and secretion titer increased with buffer and salt concentration (Fig. 3a, Additional file 1: Figure S5). Plotting relative expression and secretion titer against conductivity revealed a linear relationship, irrespective of species (Fig. 3b, Additional file 1: Figure S5). The correlation was stronger for secretion titer (R^2^ = 0.8) than expression (R^2^ = 0.7). The highest concentrations of buffers and salts resulted in only a twofold relative increase in expression and secretion titer compared to the nearly six-fold increase observed in media with a combination of phosphate and glycerol (Fig. 1b), indicating that high ionic content is but one component of an optimal medium formulation.

### Carbon sources and buffers have a synergistic effect on expression and secretion titer

Independent addition of buffers, salts and carbon sources showed that secretion titer increases with ionic content and decreases with carbon sources that cause acidification of the culture. If acidification of the culture were the sole cause of decreased secretion titer, addition of a buffer would return secretion titer to levels at least equivalent to no added carbon source. Potassium phosphate and glycerol in LB-L had a synergistic effect, however, suggesting that high ionic strength, buffering, and added carbon sources are a critical combination for increased secretion titer.

To determine if the synergistic effect could be a result of any combination of carbon source and buffer at high ionic strength or whether the effect was specific to the combination of potassium phosphate and glycerol, we measured secretion of SptP-DH in LB media containing potassium phosphate, MOPS, sodium chloride, and MOPS plus sodium chloride with added glucose or glycerol. We chose these two carbon sources because glucose had a strong negative effect on SptP-DH expression and secretion titer, while glycerol alone had no effect on SptP-DH expression, a moderate negative effect on secretion titer, and in combination with phosphate buffer caused a significant increase in expression and secretion titer (Fig. 1, Additional file 1: Figure S1). In addition to representing the spectrum of effects observed in this study, glucose and glycerol are prominent carbon sources in industry and in research studies [26–28]. The potassium phosphate and MOPS concentrations were 90 mM to match the concentration of potassium phosphate in TB, and the concentrations of sodium chloride with and without MOPS buffer were chosen to approximate the conductivity of 90 mM potassium phosphate (Additional file 1: Table S7). Expression and secretion titer of SptP-DH were measured in comparison to LB-L with no added carbon source using semi-quantitative western blotting.

**Fig. 4:**
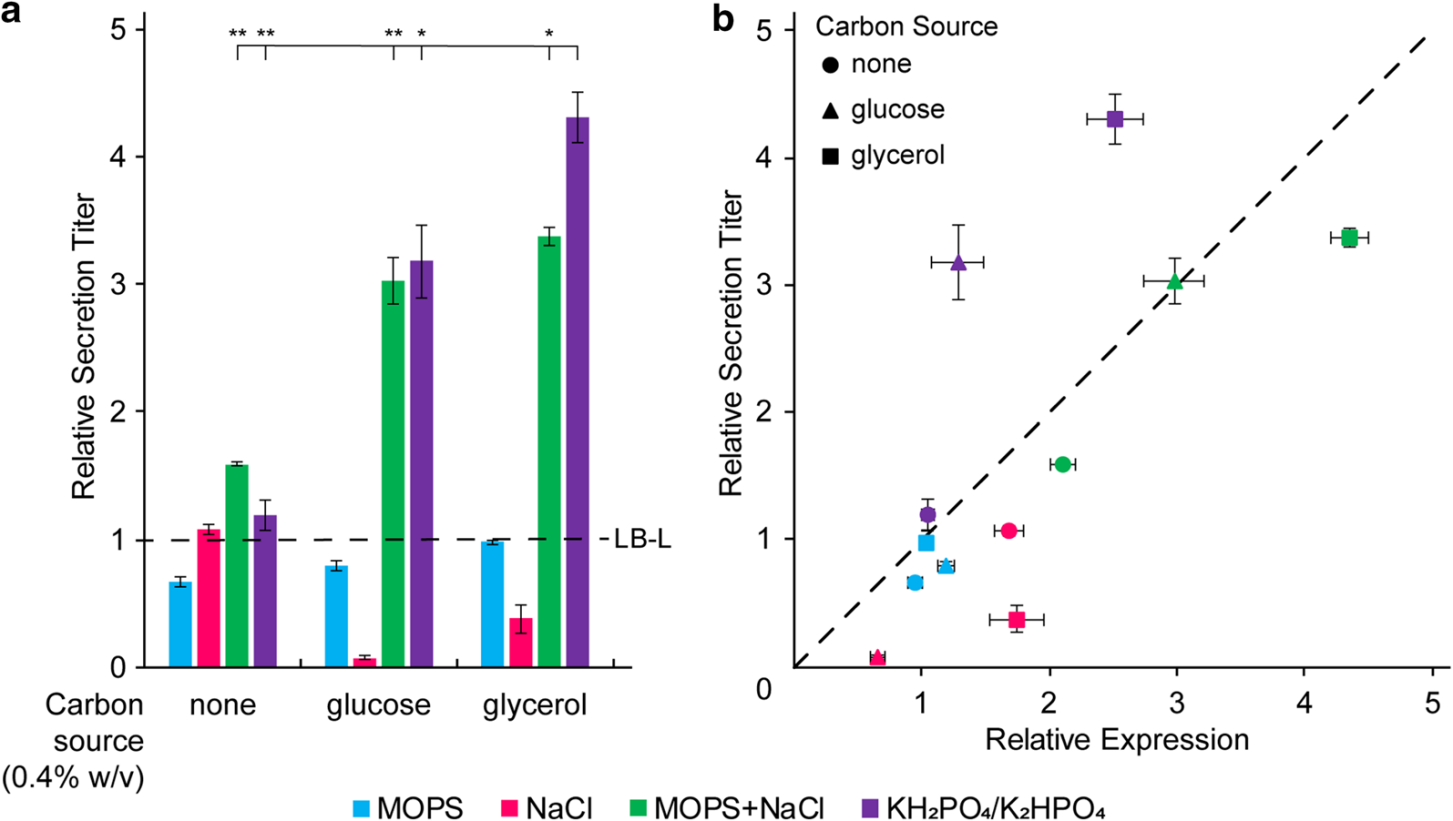
Sugars and buffers have a synergistic effect on expression and secretion titer. **a** Relative bulk secretion titer of SptP-DH-2xFLAG-6xHis secreted in media containing 10 g/L tryptone, 5 g/L yeast extract, and the additives listed in Additional file 1: Table S7. Bulk secretion titer was normalized to LB-L with no additives (dotted line) using semi-quantitative western blotting. Statistical significance was determined by Welch’s unequal variances t-test. Significance denoted by “**” represents p < 0.01, and “*” represents p < 0.05. **b** Relative bulk secretion titer versus relative bulk expression. All error bars represent standard error for three biological replicates

Buffering was essential to maintain secretion in the presence of glucose and glycerol, and the combination of buffering and increased ionic content was necessary to increase expression and secretion titer (Fig. 4a, b). LB-MOPS with glucose or glycerol produced secretion titers similar to LB-L with no added carbon source, while secretion titer decreased in LB-NaCl with glucose or glycerol. The decrease in secretion titer in LB-NaCl with glucose or glycerol was accompanied by acidification of the extracellular environment, as observed in Fig. 2 (Additional file 1: Table S7). Both LB-(KH_2_PO_4_/K_2_HPO_4_) and LB-(MOPS + NaCl) with added glucose or glycerol provided at least a threefold increase in secretion titer. LB-(MOPS + NaCl) appeared to increase secretion titer primarily by increasing expression, while LB-(KH_2_PO_4_/K_2_HPO_4_) increased expression but also increased secretion efficiency (Fig. 4b, Additional file 1: Figure S6). LB-(KH_2_PO_4_/K_2_HPO_4_) with glycerol had a specific beneficial effect—the increase in secretion titer was statistically higher than the other combinations of carbon source, buffer, and salt (p < 0.05, Fig. 4a).

### Carbon sources and buffers affect T3SS transcriptional activity

**Fig. 5:**
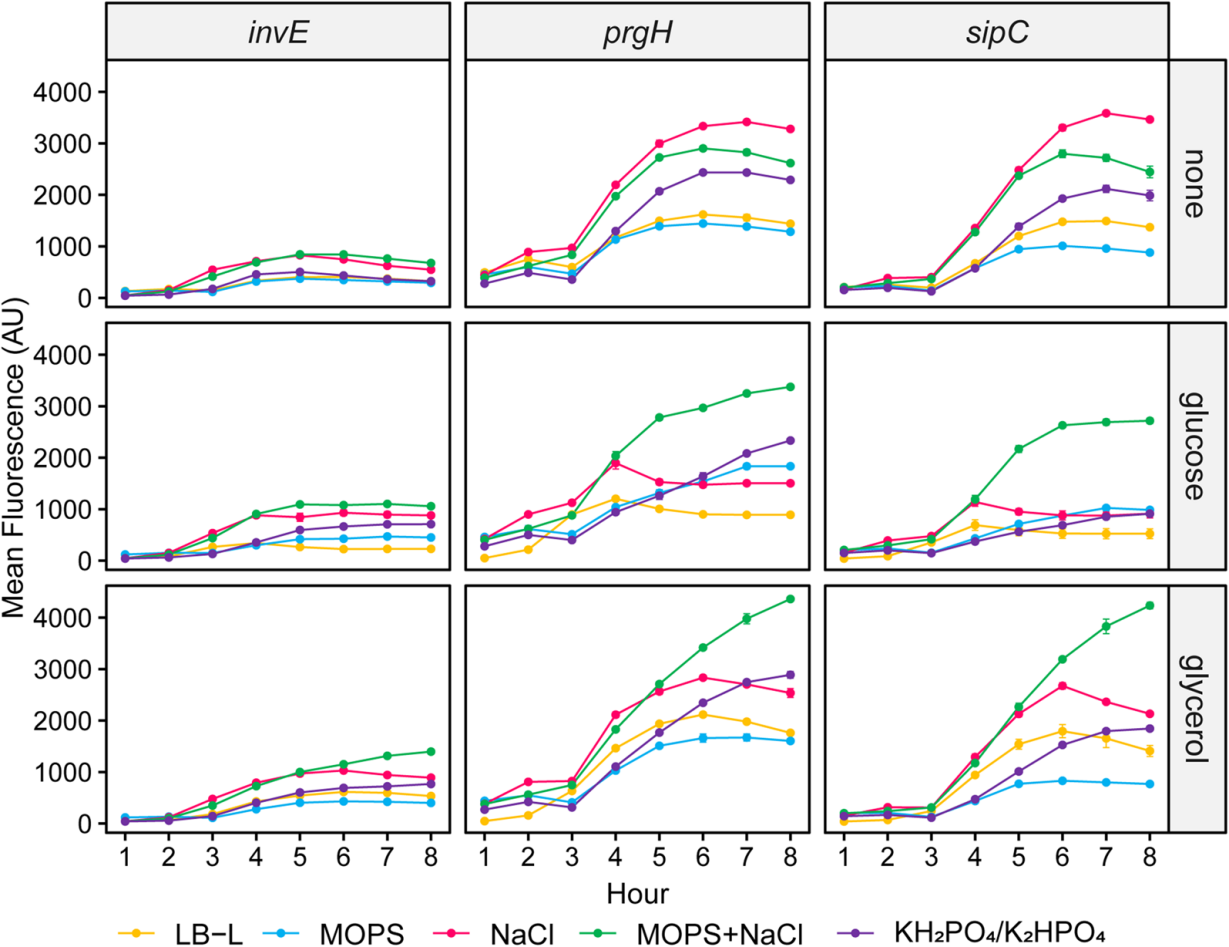
Carbon sources and buffers affect T3SS transcriptional activity. Transcriptional activity at the *inv, prg,* and *sic* loci for no added carbon source (“none”), glucose, and glycerol in the media listed in Additional file 1: Table S7 was measured over time by performing flow cytometry on ASTE13 strains containing *invE*:*gfpmut2*, *prgH*:*gfpmut2*, or *sipC*:*gfpmut2* transcriptional fusions. Error bars represent standard error of the GFP geometric mean (“Mean Fluorescence”) for three biological replicates

Flow cytometry performed on ASTE13 strains containing transcriptional fusions of GFP at the *sic, prg*, and *inv* loci in the media listed in Additional file 1: Table S7 showed that SPI-1 transcriptional activity varied with medium composition (Fig. 5). Transcriptional activity increased with conductivity for no added carbon source. In media containing glucose or glycerol, the trends were more complex. Transcriptional activity was repressed in LB-NaCl and highest in LB-(MOPS + NaCl). LB-(KH_2_PO_4_/K_2_HPO_4_) with glucose or glycerol caused higher transcriptional activity at the *prg* operon than either the *inv* or *sic* operons. Relative to media with no added carbon source, glucose and glycerol also prolonged transcriptional activation in buffered media while causing an early decrease in unbuffered media. Histograms for the data in Fig. 5 are available in Additional file 1: Figures S7–S9.

We again compared maximum transcriptional activity and duration of T3SS activation to expression and secretion titer on a per cell basis, as we did for the data in Fig. 2. Expression per cell corresponded more strongly with maximum transcriptional activity (Additional file 1: Figure S10A), while secretion per cell corresponded more strongly with prolonged activation of T3SS loci (Additional file 1: Figure S10B). Again, the strength of the correlations varied by locus and parameter. Expression and secretion per cell showed the strongest correlation with maximum mean fluorescence at the *prgH* locus, and length of activation at the *sipC* locus had the strongest correlation with secretion and expression per cell. Neither transcriptional activity nor dynamics at the *invE* locus correlated well with expression or secretion titer per cell.

### Increases in secretion titer from optimized growth media and strain improvements are additive and general for diverse secreted proteins

In prior work, we showed that knocking out a protein in the SPI-1 T3SS tip complex, SipD, increased secretion titer twofold in strains with *hilA* overexpression [29]. An ideal T3SS production platform would combine all features that increase secretion titer, so we evaluated if *∆sipD* was additive with an optimized medium. To determine if the effect was general, we selected a variety of test proteins in addition to DH: magainin-1 (MAG1), an antimicrobial peptide; 14B7*, an scFv against the protective antigen of the anthrax toxin [30, 31]; and recombinant human growth hormone (rhGH, mature somatropin). All proteins were cloned in the format SptP-POI-2xFLAG-6xHis and secreted in ASTE13 WT and *∆sipD* strains. We added 90 mM NaCl to LB-KH_2_PO_4_/K_2_HPO_4_ with glycerol to match the conductivity of LB-L supplemented with glycerol and potassium phosphate (Additional file 1: Table S4). We will refer to this medium as LB-ES for “enhanced secretion”.

**Fig. 6:**
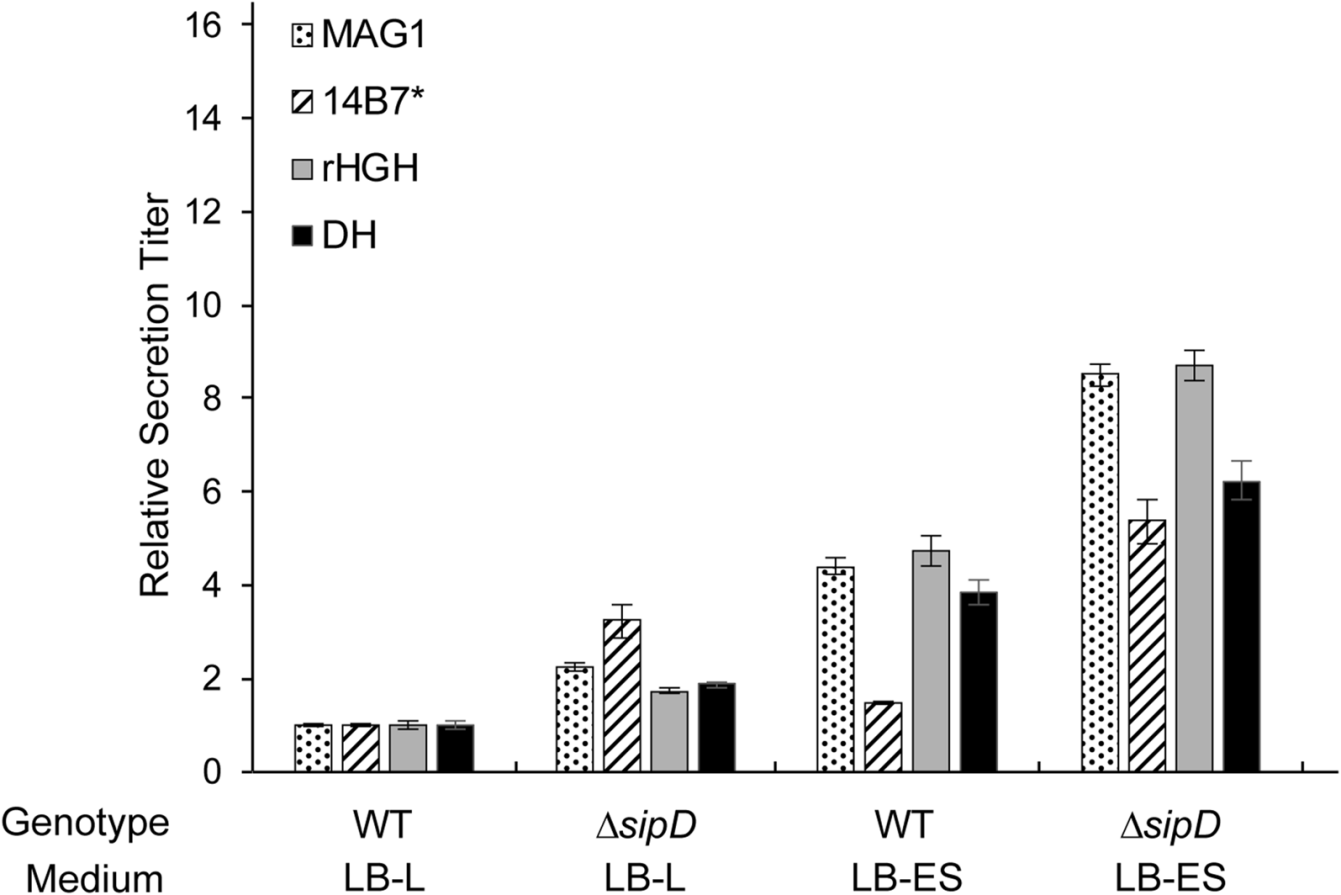
Increases in secretion titer from optimized media and strain modifications are additive. Relative bulk secretion titer of test proteins from WT and *∆sipD* strains in LB-L or LB-ES media. “LB-ES” is 10 g/L tryptone, 5 g/L yeast extract, 80 mM KH_2_PO_4_/K_2_HPO_4_ pH 7.4, 90 mM NaCl, and 0.4% w/v glycerol. Bulk relative secretion titer was normalized to ASTE13 WT in LB-L for each protein using semi-quantitative western blotting. All error bars represent standard error for three biological replicates

The *∆sipD* strain improvement and the optimized medium were indeed additive for all proteins tested (Fig. 6, Additional file 1: Figure S11). Secretion titer increased by varying amounts, but the minimum increase provided by the combination of *∆sipD* and LB-ES was six-fold above a WT strain in LB-L. Total protein expression showed a different pattern from secretion titer. SptP-MAG1 and SptP-DH followed similar expression patterns, and the apparent effect of *∆sipD* and LB-ES was to increase secretion efficiency for these proteins. SptP-rhGH and SptP-14B7* expression increased by a surprising 8- and 14-fold, however. SptP-rhGH expression increased by the same fraction as secretion titer in each condition, suggesting that secretion efficiency remained constant. SptP-14B7* secretion titer increased by a much smaller margin than expression in both WT and *∆sipD* strains with LB-ES. We hypothesize that the discrepancy was caused by loss of expressed protein to insoluble aggregates, preventing secretion.

## Discussion

Heterologous protein secretion in bacteria retains the robust, low-cost potential of bacterial protein production while significantly decreasing the complexity of the product purification process. The secreted fraction contains fewer biomolecules than lysate, which allows recovery of the purified product in a single step [4]. Here we expanded our efforts to engineer the *S. enterica* SPI-1 T3SS as a heterologous protein production platform by identifying and optimizing medium components that promoted high secretion titers. The combination of high ionic content, buffering capacity, and a glycolytic carbon source was critical for increased secretion titer. A mixture of phosphate buffer, sodium chloride, and glycerol fulfilled this requirement and provided titers of 10 ± 1 mg/L for the antimicrobial peptide magainin-1, 1 ± 0.3 mg/L for the scFv 14B7*, and 25 ± 3 mg/L for human growth hormone (Additional file 1: Figure S12).

Increased secretion titer correlated with increased expression, though secretion efficiency varied with medium composition. The data in Fig. 6 and Additional file 1: Figure S11A implies that that there is an upper limit for the benefit of increased expression, however, so secretion efficiency is a more desirable optimization target. Maximizing secretion efficiency—finding the optimum between expression and secretion titer that minimizes product loss to insoluble aggregates and maximizes the fraction of expressed protein that is secreted—will optimize use of cellular resources. The T3SS is a ~ 4 MDa structure, so optimizing use of cellular resources is critical to create an efficient cellular reactor with selectivity for T3SS assembly, heterologous protein expression, and T3SS operation for heterologous protein secretion.

Secretion titer increased with the length of secretion system transcriptional activation, while expression correlated with maximum transcriptional activity at the *inv*, *prg*, and *sic* loci (Figs. 2, 5; Additional file 1: Figures S4, S10). Heterologous protein expression was controlled by P_*sicA*_, the native SPI-1 promoter that controls the *sic* operon, so it was not surprising that heterologous protein expression correlated with maximum transcriptional activity. The limited benefit of increased heterologous protein expression suggests that prolonging secretion system activation is a more efficient use of cellular resources than maximizing transcriptional activity to increase secretion titer. Carbon sources increased the length of secretion system activation, but only in the presence of sufficient buffering. Acidification of the medium to a pH below 6.0 at harvest coincided with an early arrest of transcriptional activity at all the T3SS loci, and it had an even stronger negative effect on secretion titer (Figs. 2, 5; Additional file 1: Figures S4, S10). Secretion via the T3SS requires the proton motive force (PMF) [32], so it is possible that acidification of the extracellular environment deactivated the secretion apparatus.

Though expression and secretion titer increased with medium conductivity, a proxy for ionic content, conductivity was insufficient to fully explain the effect of medium composition. High conductivity and buffering in the presence of a carbon source increased secretion titer, but the identity of the buffer and the carbon source were important for the observed effect. It is also likely that other species present in the undefined tryptone and yeast extract affect secretion titer. 2X YT and LB-(MOPS + NaCl) or LB-(KH_2_PO_4_/K_2_HPO_4_) with glucose provided similar increases in expression and secretion titer, but the compositions of those media were quite different. A systematic analysis of the effects of the chemical identity and concentration of the components of tryptone and yeast extract on expression and secretion titer would more clearly identify the critical combination of ingredients for a growth medium that produces robust and high secretion titers. A fully chemically defined media would facilitate that investigation.

Medium design is a critical aspect of any protein production process, but it is especially important for heterologous protein production via the SPI-1 T3SS because of the sensitivity of the T3SS to the extracellular environment. The regulation that governs that sensitivity is multilayered and interwoven [15–20], but the results of this study suggest we can positively control T3SS function through traditional medium optimization strategies.

## Conclusion

In this study, we optimized the extracellular environment to promote protein secretion via the *S. enterica* T3SS. We explored the individual effects of carbon sources, buffering agents, and salts on secretion titer and discovered that a combination of the three elements was synergistic. An optimized combination of glycerol, phosphate, and sodium chloride in a rich nutrient base increased secretion titer at least fourfold for a variety of secreted proteins. Analyzing the connection between transcriptional activity, heterologous protein expression, and secretion titer revealed that prolonging secretion system activation is key to achieving high secretion titers. With this knowledge, we can design a fully chemically defined medium and precisely optimize other environmental factors, such as dissolved oxygen and controlled growth via fed-batch or continuous culture strategies. Taking advantage of native T3SS regulation to define an extracellular environment that promotes robust, high secretion titers will identify new regulatory mechanisms and advance the development of the T3SS as a heterologous protein production platform.

## Methods

### Strains and growth conditions

Strains and plasmids used in this work are listed in Additional file 1: Tables S1, S2. *S. enterica* Typhimurium ASTE13 is a derivative of *S. enterica* Typhimurium DW01, which is itself a derivative of *S. enterica* Typhimurium LT2 [33]. Secretion experiments were started by growing a single colony from a fresh streak of a frozen glycerol stock in the lysogeny broth Lennox formulation (10 g/L tryptone, 5 g/L yeast extract, 5 g/L NaCl) with appropriate antibiotics (34 μg/mL chloramphenicol, 50 μg/mL kanamycin) for 12–16 h overnight in an orbital shaker at 37 °C and 225 rpm. Overnight cultures were diluted 1:100 into the appropriate medium supplemented with 100 μg/mL isopropyl β-d-1-thiogalactopyranoside (IPTG) and appropriate antibiotics. All culturing steps were performed in 24-well deepwell plates (Axygen). Secretion was allowed to proceed for 8 h following induction at 37 °C and 225 rpm in an orbital shaker. Whole culture lysate samples for SDS-PAGE were prepared by adding cell suspension to Laemmli buffer [34] at the end of secretion.

We centrifuged the cultures at 4000×*g* for 10 minutes and collected the supernatant; this supernatant is what we call the secretion fraction. SDS-PAGE samples were prepared by mixing the secretion fraction with Laemmli buffer. To stay within the linear range on each western blot, secretion fractions with secreted protein titers higher than the control condition (LB-L with no additives) were further diluted 2, 4, or 8 times in 1X Laemmli buffer. All SDS-PAGE samples were boiled at 95 °C for 5 min immediately after preparation.

### Medium formulations

“LB” refers to a base medium formulation of 10 g/L tryptone and 5 g/L yeast extract, and “TB” is the standard Terrific Broth formulation: 12 g/L tryptone, 24 g/L yeast extract, 9.4 g/L K_2_HPO_4_, 2.2 g/L KH_2_PO_4_, and 0.4% w/v glycerol. LB-L is 10 g/L tryptone, 5 g/L yeast extract, and 5 g/L NaCl. Carbon sources were prepared as 20% w/v solutions, sterile filtered, and diluted into the medium to final concentrations of 0.4% w/v at the time of subculture. Media with buffers and salts were formulated by autoclaving LB medium at a 1.2X concentration and adding the appropriate volumes of 1 M K_2_HPO_4_, 1 M KH_2_PO_4_, 1 M MOPS, or 5 M NaCl. Ultrafiltered water was added as necessary to achieve a final 1X LB concentration. The pH of all media was adjusted to 7.4 using HCl or NaOH as appropriate. Tryptone and yeast extract were sourced from BD Bacto. Conductivity and pH were measured using a Fisher Scientific accumet AB150 meter.

### Plasmid construction

Golden Gate cloning was used to construct plasmids for this study [35]. Genes for proteins to be secreted were inserted into a modified pPROTet.133 backbone vector (BD Clontech) under the control of the *sic* promoter [7]. All secretion plasmids expressed the SptP chaperone *sicP* and the *sptP* secretion signal (nucleotides 1 to 477). The SptP secretion tag was fused N-terminal to the protein of interest, and 2xFLAG and 6xHis tags were fused C-terminal to the protein of interest to facilitate purification and immunodetection. The gene for rhGH was ordered from Twist Biosciences with overhangs compatible with Golden Gate cloning. PCR was performed with Phusion DNA polymerase to amplify the MAG1 gene using the primers listed in Additional file 1: Table S3. We used *E. coli* DH10B cells for all cloning, and all DNA sequences were confirmed by Sanger sequencing (Quintara).

### Recombineering

Recombineering was performed in *S. enterica* Typhimurium ASTE13 as described by Thomason et al*.* [36]. Briefly, a *cat-sacB* cassette conferring chloramphenicol resistance and sucrose sensitivity was amplified from the *E. coli* TUC01 genome using primers with 40 bp of homology 5′ and 3′ to the locus of interest. The *GFPmut2* gene was amplified using primers containing the same 40 bp of homology 5′ and 3′ to the locus of interest as used for the *cat-sacB* cassette. PCR was performed with Phusion DNA polymerase. *S. enterica* Typhimurium ASTE13 was first transformed with pSIM6. A first round of recombineering was performed to insert the *cat-sacB* cassette at the locus of interest, and a second round of recombineering replaced the *cat-sacB* cassette with an appropriate DNA product. The replacement DNA was *GFPmut2* for transcriptional fusions and a 200 bp double-stranded PCR product containing the first and last 30 bp of *sipD* flanked 5′ and 3′ by 70 bp of homology to the *sipD* genetic locus. The genomic modifications were confirmed by Sanger sequencing (Quintara), and the strains were cured of pSIM6. Primers used for recombineering are listed in Additional file 1: Table S3.

### Protein separation and western blotting

Samples were separated by SDS-PAGE and transferred to a polyvinylidene fluoride membrane (PVDF, Millipore) for western blotting using the Bio-Rad Criterion blotter. Samples were diluted such that all band signals were within twofold of the average signal across the blot. Membranes were probed with mouse anti-FLAG per manufacturer’s instructions (Sigma Aldrich). To facilitate chemiluminescent detection, a secondary labeling step was performed with goat anti-mouse IgG (H+L) HRP conjugate according to manufacturer’s instructions (Thermo Fisher). Bands were developed with the SuperSignal West Pico Plus substrate (Thermo Fisher) according to manufacturer’s instructions and imaged with ChemiDoc XRS + imaging system (Bio-Rad).

For anti-GroEL blotting, membranes used for anti-FLAG blotting were stripped and re-probed using a protocol derived from Abcam. Briefly, membranes were incubated with mild stripping buffer (15 g/L glycine, 1 g/L SDS, 1% v/v Tween 20, pH 2.2) for two periods of five minutes, washed with water for two ten-minute periods, washed with TBST for two five-minute periods, and then blocked with 5% w/v nonfat milk (Research Products International) dissolved in TBST for one hour. Membranes were probed with rabbit anti-GroEL (Millipore Sigma) according to manufacturer’s instructions. Membranes were subsequently labeled with goat anti-rabbit IgG (H + L) conjugated to HRP according to manufacturer’s instructions. Bands were detected with the SuperSignal West Pico Plus substrate (Thermo Fisher) and a ChemiDoc XRS + imaging system.

### Protein quantification

All relative protein quantities were calculated by performing densitometry using Image Lab software (Bio-Rad) and normalizing to the average of the replicates of the specified normalization condition. Relative protein amounts were corrected for dilution if appropriate. Absolute secretion titers were measured by performing SDS-PAGE, staining with Coomassie according to Studier [37], and calculating densitometry relative to a bovine serum albumin standard curve (Thermo Scientific Pierce). Background was calculated by averaging the signal at the same molecular weight as the protein of interest across all other lanes containing secreted fractions and then subtracted from the signal of the protein of interest. Error bars are standard deviation on three biological replicates unless otherwise specified.

### Flow cytometry

ASTE13 strains carrying *invE:gfpmut2*, *prgH:gfpmut2*, or *sipC:gfpmut2* transcriptional fusions and P_*lacUV5*_* hilA* were grown and induced as specified in “Strains and Growth Conditions”. Samples were prepared by diluting cultures to an optical density at 600 nm of 0.005 to 0.05 in PBS with 1 mg/mL kanamycin in round-bottom 96-well plates (Greiner Bio-One #650101). Plates were sealed and stored at 4 °C for analysis. For each sample, an Attune NxT flow cytometer (Life Technologies) was used to collect 10,000 events within a gated population determined to be cells. Data were analyzed using FlowJo 10.5.3 (TreeStar, Inc.). The experiment was performed in biological triplicate, and error bars represent standard error. Spearman correlations were calculated and plotted in R.

## Supplementary Information

**Additional file 1: Table S1.** Strains used in this study. **Table S2.** Plasmids used in this study. **Table S3.** Primers used in this study. **Figure S1. A** Relative expression (*i*) and representative western blots (*ii*) of SptP-DH-2xFLAG-6xHis in LB-L, 2X YT, and TB. **B** Relative expression (*i*) and representative western blots (*ii*) of SptP-DH-2xFLAG-6xHis in LB-L supplemented with the defined components in TB. All western blots are representative of four biological replicates. Samples were diluted to fall within the linear range of the LB-L signal. Boxed bands are from the same blot but were rearranged for clarity. “WCL” is whole culture lysate and “SF” is secreted fraction. Error bars represent standard error of the mean for four biological replicates. **Table S4.** Conductivity of media at start of experiment and OD_600nm_ and secreted fraction pH at time of harvest. **Figure S2.** Western blots representative of three biological replicates for LB-L supplemented with various carbon sources. “WCL” is whole culture lysate and “SF” is secreted fraction. **Table S5.** OD_600nm_ and secreted fraction pH at time of harvest for LB-L supplemented with various carbon sources. **Figure S3.** Representative histograms of flow cytometry GFP signal for SPI-1 transcriptional activity in LB-L with 0.4% w/v carbon sources (Fig. 2). Overlaid populations represent hourly time points from 1 h (red) to 8 h (teal). “ASTE13 WT” is a negative control. **Figure S4. A** Spearman correlations between expression or secretion per cell and hour at maximum mean fluorescence from flow cytometry in Fig. 2. **B** Spearman correlations between expression or secretion per cell and maximum mean fluorescence from flow cytometry in Fig. 2. Expression and secretion per cell were calculated by dividing densitometry by OD_600nm_ and normalizing to LB-L with no additives. Each data point is a replicate. **Table S6.** Medium additives to evaluate the effect of phosphate buffer on expression and secretion titer. Species in each “level” were designed to have a similar buffer concentration and/or conductivity to the corresponding concentration of KH_2_PO_4_/K_2_HPO_4_. **Figure S5. A** Relative bulk expression of SptP-DH-2xFLAG in LB supplemented with the buffers and salts listed below the graph. Species in each “level” were designed to have a similar buffer concentration (MOPS, MOPS + NaCl) and/or conductivity (NaCl, MOPS + NaCl) to the corresponding concentration of KH_2_PO_4_/K_2_HPO_4_. Each column of the table corresponds to the group of bars above it, and each row of the column corresponds to a separate bar in the group. Data was normalized to LB-L using semi-quantitative western blotting. Error bars represent standard error for four biological replicates except for black-outlined bars, which represent six biological replicates. LB-L and LB-IM are denoted by dashed lines. **B** Relative bulk expression versus conductivity for the media in part **A.** The gray shading represents a 95% CI on the linear regression. Each data point is a replicate except for LB-L, which is labeled as a reference. **C** Western blots representative of four biological replicates. “WCL” is whole culture lysate and “SF” is secreted fraction. **Table S7.** Conductivity of media at start of experiment and OD_600nm_ and secreted fraction pH at time of harvest for LB supplemented with buffers, salts, and carbon sources. **Figure S6. A** Relative bulk expression of SptP-DH-2xFLAG-6xHis in media containing 10 g/L tryptone, 5 g/L yeast extract, and the additives listed in Table S7. Bulk expression was normalized to LB-L with no additives (dotted line). Error bars represent standard error of the mean for three biological replicates. **B** Western blots are representative of three biological replicates. Samples were diluted to fall within the linear range of the LB-L signal. Boxed bands are from the same blot but were rearranged for clarity. “WCL” is whole culture lysate and “SF” is secreted fraction. **Figure S7**. Representative histograms of GFP signal for flow cytometry to measure SPI-1 transcriptional activity in the media listed in Table S7 with no added carbon source (“none”; Fig. 5). Overlaid populations represent hourly time points from 1 h (red) to 8 h (teal). “ASTE13 WT” is a negative control. **Figure S8.** Representative histograms of GFP signal for flow cytometry to measure SPI-1 transcriptional activity in the media listed in Table S7 with 0.4% w/v glucose (“glucose”; Fig. 5). Overlaid populations represent hourly time points from 1 h (red) to 8 h (teal). “ASTE13 WT” is a negative control. **Figure S9.** Representative histograms of GFP signal for flow cytometry to measure SPI-1 transcriptional activity in the media listed in Table S7 with 0.4% w/v glycerol (“glycerol”; Fig. 5). Overlaid populations represent hourly time points from 1 h (red) to 8 h (teal). “ASTE13 WT” is a negative control. **Figure S10. A** Spearman correlations between expression or secretion per cell and hour at maximum mean fluorescence from flow cytometry in Fig. 5. **B** Spearman correlations between expression or secretion per cell and maximum mean fluorescence from flow cytometry in Fig. 5. Expression and secretion per cell were calculated by dividing densitometry by OD_600nm_ and normalizing to LB-L with no additives. Each data point is a replicate. **Figure S11. A** Relative bulk expression of test proteins from WT and *∆sipD* strains in LB-L or LB-ES media. “LB-ES” is 10 g/L tryptone, 5 g/L yeast extract, 80 mM KH_2_PO_4_/K_2_HPO_4_ pH 7.4, 90 mM NaCl, and 0.4% w/v glycerol. Relative bulk expression was normalized to secretion from ASTE13 WT in LB-L for each protein using semi-quantitative western blotting. Error bars represent standard error of the mean for three biological replicates. **B** Western blots are representative of three biological replicates. Samples were diluted to fall within the linear range of the normalization signal. Boxed bands are from the same blot but were rearranged for clarity. “WCL” is whole culture lysate and “SF” is secreted fraction. **Figure S12.** Titer of secreted proteins.

## Data Availability

All data generated and/or analyzed during this study is included in the published article and its additional file.

## Abbreviations

LB: Lysogeny broth
POI: Protein of interest
MOPS: 3-(*N*-Morpholino)propanesulfonic acid
scFv: Single chain variable fragment
SPI-1: *Salmonella* pathogenicity island 1
T3SS: Type III secretion system
TB: Terrific broth
