# Supplementary material for: An optimized growth medium for increased recombinant protein secretion titer via the type III secretion system
Source: Microb Cell Fact. 2021 Feb 15;20:44. doi: 10.1186/s12934-021-01536-z (PMC7885374; doi:10.1186/s12934-021-01536-z)
Supplement: Supplementary file 1 — Additional file 1: Table S1. Strains used in this study. Table S2. Plasmids used in this study. Table S3. Primers used in this study. Figure S1. A Relative expression (i) and representative western blots (ii) of SptP-DH-2xFLAG-6xHis in LB-L, 2X YT, and TB. B Relative expression (i) and representative western blots (ii) of SptP-DH-2xFLAG-6xHis in LB-L supplemented with the defined components in TB. All western blots are representative of four biological replicates. Samples were diluted to fall within the linear range of the LB-L signal. Boxed bands are from the same blot but were rearranged for clarity. “WCL” is whole culture lysate and “SF” is secreted fraction. Error bars represent standard error of the mean for four biological replicates. Table S4. Conductivity of media at start of experiment and OD600nm and secreted fraction pH at time of harvest. Figure S2. Western blots representative of three biological replicates for LB-L supplemented with various carbon sources. “WCL” is whole culture lysate and “SF” is secreted fraction. Table S5. OD600nm and secreted fraction pH at time of harvest for LB-L supplemented with various carbon sources. Figure S3. Representative histograms of flow cytometry GFP signal for SPI-1 transcriptional activity in LB-L with 0.4% w/v carbon sources (Fig. 2). Overlaid populations represent hourly time points from 1 h (red) to 8 h (teal). “ASTE13 WT” is a negative control. Figure S4. A Spearman correlations between expression or secretion per cell and hour at maximum mean fluorescence from flow cytometry in Fig. 2. B Spearman correlations between expression or secretion per cell and maximum mean fluorescence from flow cytometry in Fig. 2. Expression and secretion per cell were calculated by dividing densitometry by OD600nm and normalizing to LB-L with no additives. Each data point is a replicate. Table S6. Medium additives to evaluate the effect of phosphate buffer on expression and secretion titer. Species in each “level” were designed to [file 12934_2021_1536_MOESM1_ESM.docx]

# ADDITIONAL INFORMATION

***Title:***

An optimized growth medium for increased recombinant protein secretion titer via the type III secretion system

***Authors:***

Lisa Ann Burdette^1†^, Han Teng Wong^2‡^, Danielle Tullman-Ercek^3*^

***Affiliations:***

1. Lisa Ann Burdette
   1. Department of Chemical and Biomolecular Engineering, University of California-Berkeley and Department of Chemical and Biological Engineering, Northwestern University
   2. lburdette@berkeley.edu
2. Han Teng Wong
   1. Department of Plant and Microbial Biology, University of California-Berkeley and Department of Chemical and Biological Engineering, Northwestern University
   2. wong_han_teng@imcb.a-star.edu.sg
3. Danielle Tullman-Ercek – Corresponding Author
   1. Department of Chemical and Biological Engineering, Northwestern University
   2. ercek@northwestern.edu

* To whom correspondence should be addressed. E-mail: ercek@northwestern.edu

†Present address: Department of Chemical and Biological Engineering, Northwestern University, Evanston, IL 60208, USA

^‡^ Present address: Institute of Molecular and Cell Biology, 61 Biopolis Way, Singapore 138673

**Table S1**. Strains used in this study.

| Strain Name | Comment | Reference |
| --- | --- | --- |
| ASTE13 | LT2-derived lab strain similar to DW01 | This study; DW01 [1] |
| ASTE13 *invE:GFPmut2* | GFPmut2 inserted immediately downstream of *invE* coding sequence | This study |
| ASTE13 *prgH:GFPmut2* | GFPmut2 inserted immediately downstream of *prgH* coding sequence | This study |
| ASTE13 s*ipC:GFPmut2* | GFPmut2 inserted immediately downstream of *sipC* coding sequence | This study |
| ASTE13 *∆sipD* | *sipD* knockout | Based on [2], newly constructed for this study |

**Table S2**. Plasmids used in this study.

| Plasmid Name | ORFs under inducible control | | ORI | ab^R^ | Reference |
| --- | --- | --- | --- | --- | --- |
| P*_sic_ DH* | *sicP* | *sptP-DH-2xFLAG-6xHis* | colE1 | cam | [3] |
| P*_sic_ MAG1* | *sicP* | *sptP-MAG1-2xFLAG-6xHis* | colE1 | cam | This study |
| P*_sic_ 14B7** | *sicP* | *sptP-14B7*-2xFLAG-6xHis* | colE1 | cam | [4] |
| P*_sic_ rhGH* | *sicP* | *sptP-rhGH-2xFLAG-6xHis* | colE1 | cam | This study |
| P*_lacUV5_ hilA* | *hilA* | | p15a | kan | [3] |

**Table S3**. Primers used in this study.

| Sequence | Amplicon | Used to Construct |
| --- | --- | --- |
| AATGGCAGAACAGCGTCGTACTATTGAAAAGCTGTCTTAA tgtgacggaagatcacttcg | *cat-sacB* | ASTE13 *invE:gfpmut2* |
| GAGAAAGCAGCACTATAGGTATCCTGTTAATATTAAA atcaaagggaaaactgtccatat | *cat-sacB* | ASTE13 *invE:gfpmut2* |
| AATGGCAGAACAGCGTCGTACTATTGAAAAGCTGTCTTAA attaaagaggagaaaggtcatgag | *gfpmut2* | ASTE13 *invE:gfpmut2* |
| GTAGAGAAAGCAGCACTATAGGTATCCTGTTAATATTAAA ttatttgtatagttcatccatgccatg | *gfpmut2* | ASTE13 *invE:gfpmut2* |
| AATGAGCCCAGGCCATTGGTATTTCCCAAGCCCACTTTAA tgtgacggaagatcacttcg | *cat-sacB* | ASTE13 *prgH:gfpmut2* |
| AAGGTGTTGCCATAATGACTTCCTTATTTACGTTAAA atcaaagggaaaactgtccatat | *cat-sacB* | ASTE13 *prgH:gfpmut2* |
| AATGAGCCCAGGCCATTGGTATTTCCCAAGCCCACTTTAA attaaagaggagaaaggtcatgag | *gfpmut2* | ASTE13 *prgH:gfpmut2* |
| ACCAAGGTGTTGCCATAATGACTTCCTTATTTACGTTAAA ttatttgtatagttcatccatgccatg | *gfpmut2* | ASTE13 *prgH:gfpmut2* |
| ATCCGCACTCGCTGCTATCGCAGGCAATATTCGCGCTTAA tgtgacggaagatcacttcg | *cat-sacB* | ASTE13 *sipC:gfpmut2* |
| AATCACACCCATGATGGCGTATAGATGACCTTTCAGA atcaaagggaaaactgtccatat | *cat-sacB* | ASTE13 *sipC:gfpmut2* |
| ATCCGCACTCGCTGCTATCGCAGGCAATATTCGCGCTTAA attaaagaggagaaaggtcatgag | *gfpmut2* | ASTE13 *sipC:gfpmut2* |
| TTAAATCACACCCATGATGGCGTATAGATGACCTTTCAGA ttatttgtatagttcatccatgccatg | *gfpmut2* | ASTE13 *sipC:gfpmut2* |
| TTTAATCGCGCTCCTGATGGCGAACTGGGGATATTATGCTTAATATTCAA tgtgacggaagatcacttcg | *cat-sacB* | ASTE13 *∆sipD* |
| CTTACACTTGTAACCATTATTAATATCCTCTTCTGTTATCCTTGCAGGAA atcaaagggaaaactgtccatat | *cat-sacB* | ASTE13 *∆sipD* |
| TCTGAAAGGTCATCTATACGCCATCATGGGTGTGATTTAATCGCGCTCCTGATGGCGAACTGGGGATATT atgcttaatattcaattcctgcaaggataa | self | ASTE13 *∆sipD* |
| TCTGCATACCTGGCATTATGACGGGGGGCTGAGTCCTTACACTTGTAACCATTATTAATATCCTCTTCTG ttatccttgcaggaattgaatattaagcat | self | ASTE13 *∆sipD* |
| aggtctcaGCTTGGCATCGGGAAATTTCTGCATAGCGCGGGAAAGTTCGGCAAAGC | MAG1 | P*_sic_ MAG1* |
| aggtctcaCGCTCGACTTCATGATCTCGCCAACAAAGGCTTTGCCGAACTTTCCCG | MAG1 | P*_sic_ MAG1* |


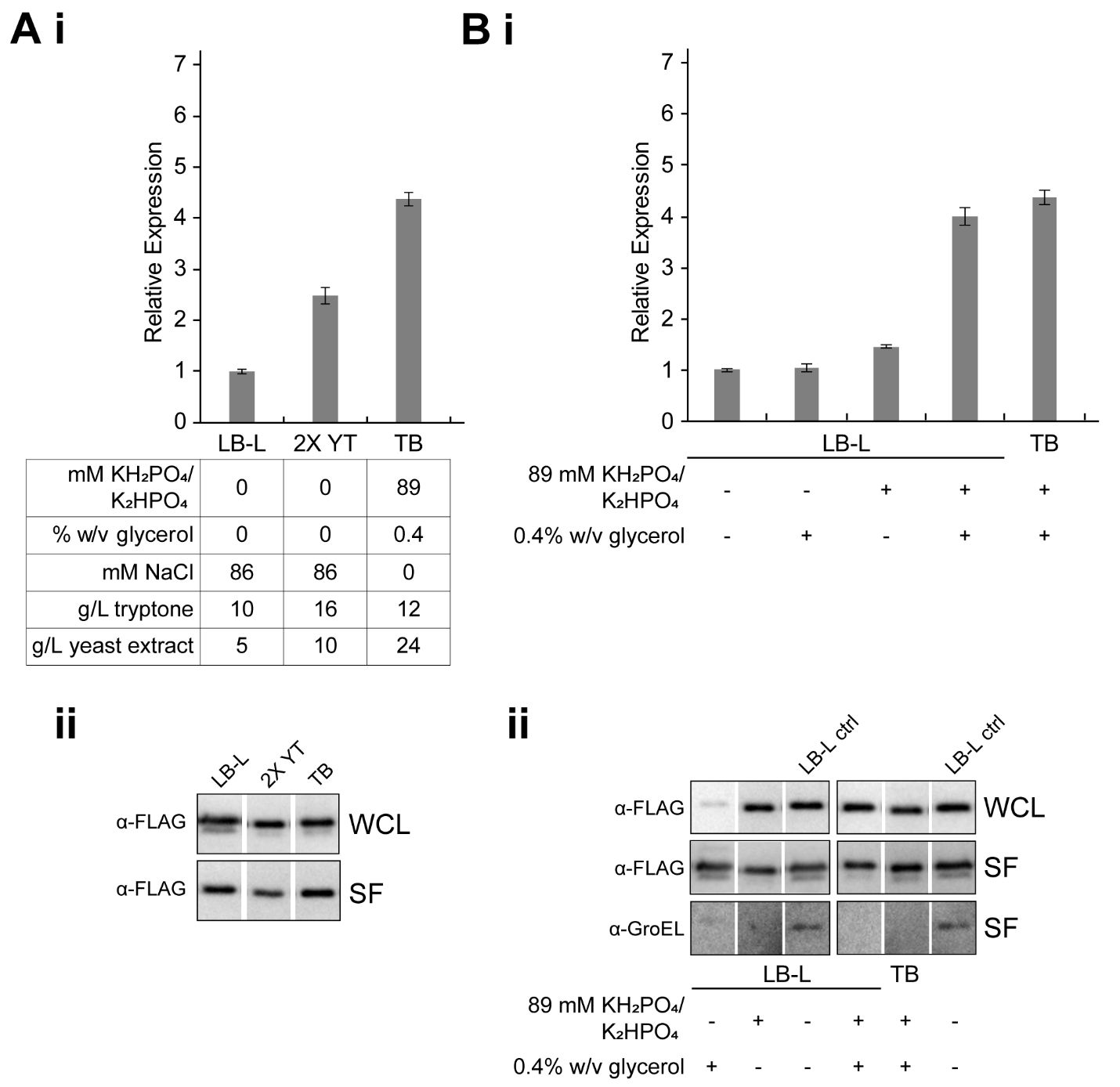


**Figure S1.** **A** Relative expression (*i*) and representative western blots (*ii*) of SptP-DH-2xFLAG-6xHis in LB-L, 2X YT, and TB. **B** Relative expression (*i*) and representative western blots (*ii*) of SptP-DH-2xFLAG-6xHis in LB-L supplemented with the defined components in TB. All western blots are representative of four biological replicates. Samples were diluted to fall within the linear range of the LB-L signal. Boxed bands are from the same blot but were rearranged for clarity. “WCL” is whole culture lysate and “SF” is secreted fraction. Error bars represent standard error of the mean for four biological replicates.

**Table S4.** Conductivity of media at start of experiment and OD_600nm_ and secreted fraction pH at time of harvest.

| Medium | [KH_2_PO_4_/  K_2_HPO_4_]  (mM) | [NaCl]  (mM) | Conductivity  (mS/cm) | pH  at 8 hr | OD_600nm_  at 8 hr |
| --- | --- | --- | --- | --- | --- |
| LB-L | 0 | 86 | 11 | 7.90 ± 0.09 | 3.43 ± 0.40 |
| 2X YT | 0 | 86 | 11 | 7.77 ± 0.03 | 3.75 ± 0.51 |
| TB | 89 | 0 | 14 | 6.55 ± 0.03 | 4.35 ± 0.78 |
| LB-L glycerol | 0 | 86 | 11 | 5.06 ± 0.03 | 3.32 ± 0.05 |
| LB-L KH_2_PO_4_/K_2_HPO_4_ | 89 | 86 | 21 | 7.37 ± 0.02 | 2.93 ± 0.02 |
| LB-L glycerol+KH_2_PO_4_/K_2_HPO_4_ | 89 | 86 | 21 | 6.62 ± 0.01 | 3.60 ± 0.09 |


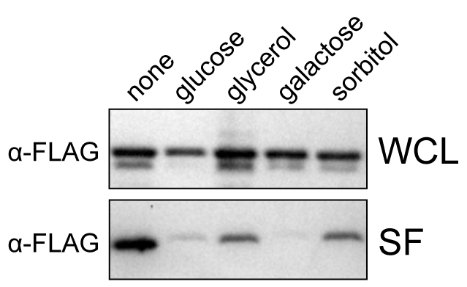


**Figure S2.** Western blots representative of three biological replicates for LB-L supplemented with various carbon sources. “WCL” is whole culture lysate and “SF” is secreted fraction.

**Table S5.** OD_600nm_ and secreted fraction pH at time of harvest for LB-L supplemented with various carbon sources.

| Carbon Source | pH  at 8 hr | OD_600nm_  at 8 hr |
| --- | --- | --- |
| None | 7.99 ± 0.10 | 3.31 ± 0.06 |
| Glucose | 4.86 ± 0.05 | 3.23 ± 0.05 |
| Glycerol | 5.14 ± 0.04 | 3.51 ± 0.17 |
| Galactose | 4.72 ± 0.01 | 3.36 ± 0.07 |
| Sorbitol | 5.15 ± 0.05 | 3.55 ± 0.13 |


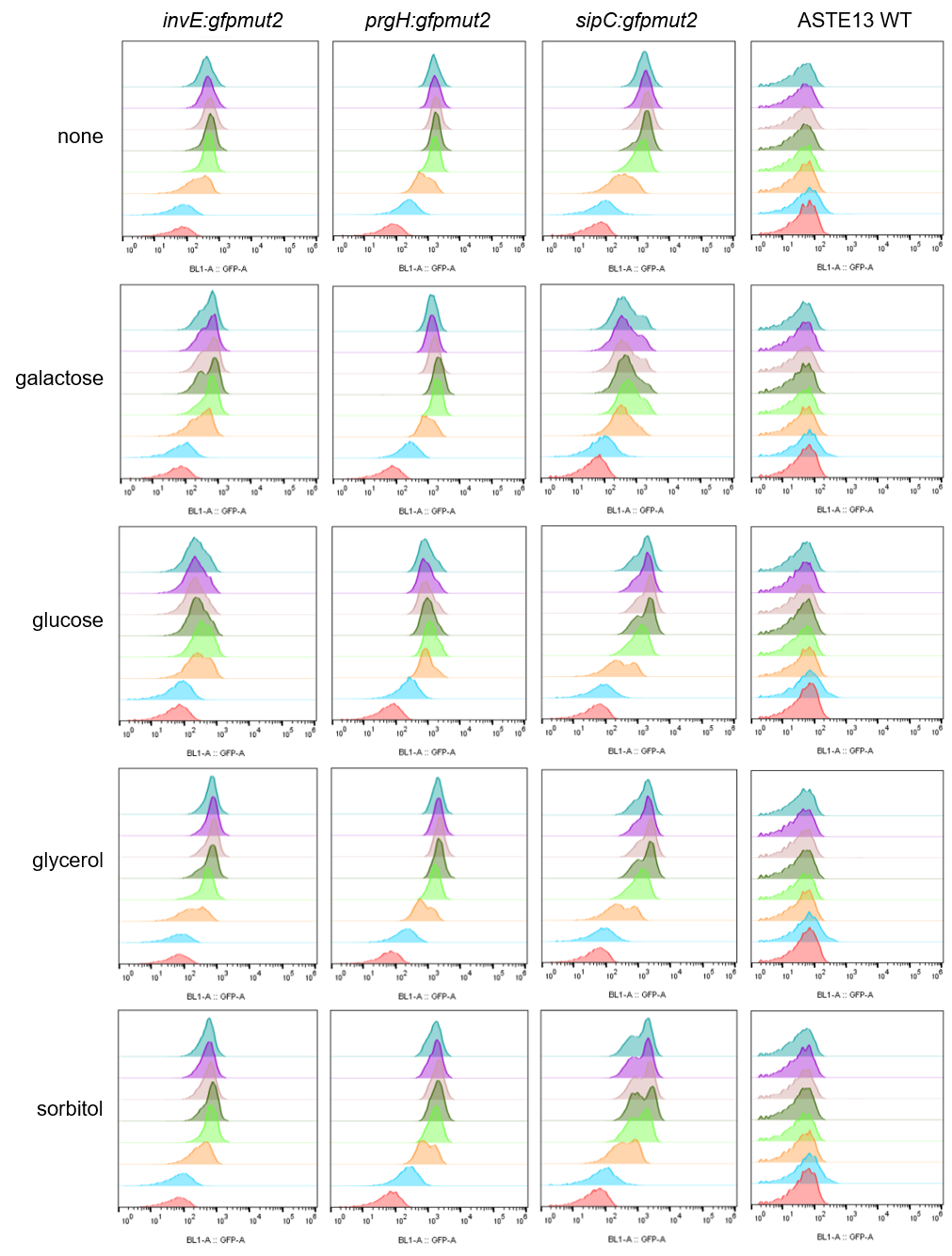


**Figure S3.** Representative histograms of flow cytometry GFP signal for SPI-1 transcriptional activity in LB-L with 0.4% w/v carbon sources (Figure 2). Overlaid populations represent hourly time points from 1 hour (red) to 8 hours (teal). “ASTE13 WT” is a negative control.


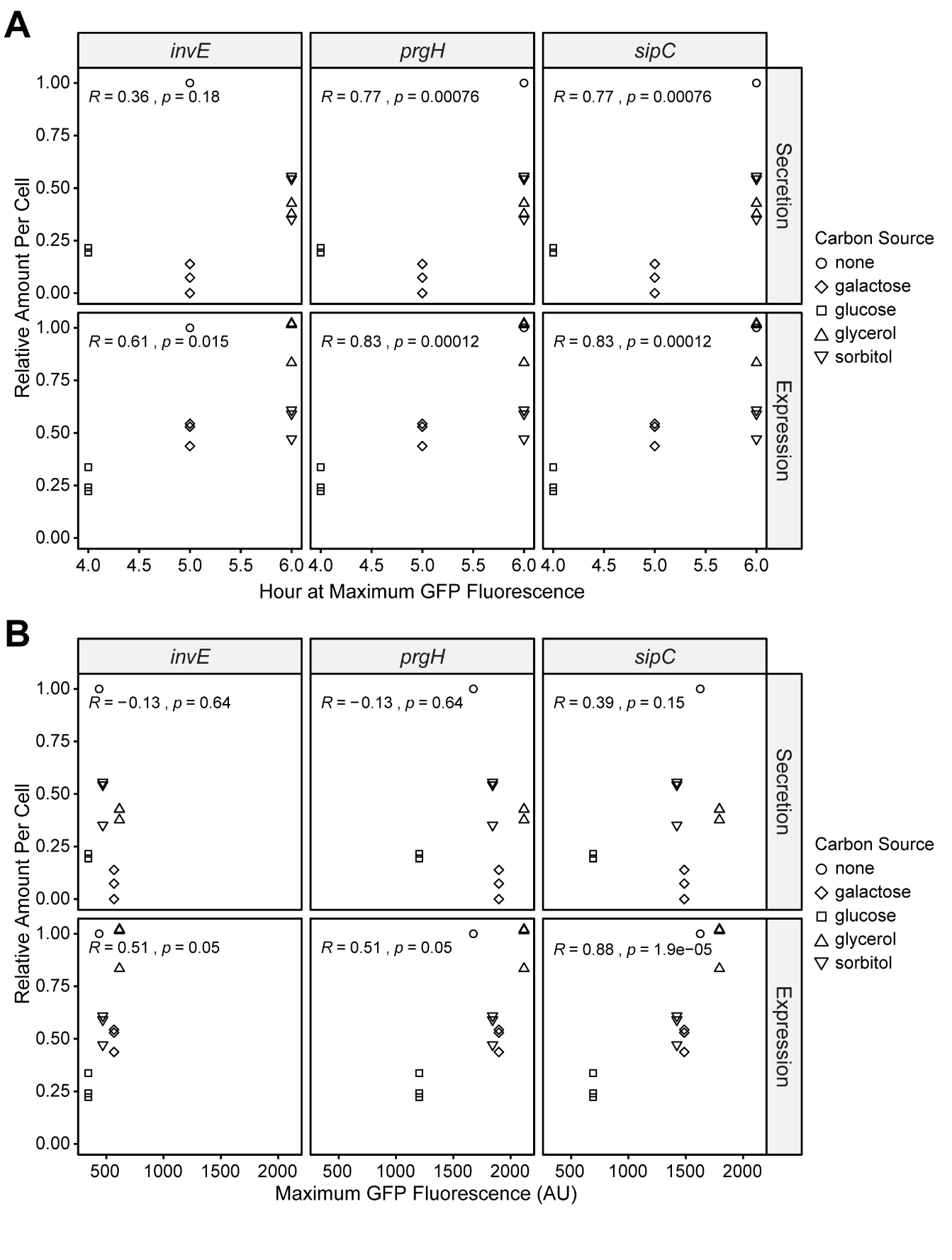


**Figure S4. A** Spearman correlations between expression or secretion per cell and hour at maximum mean fluorescence from flow cytometry in Figure 2. **B** Spearman correlations between expression or secretion per cell and maximum mean fluorescence from flow cytometry in Figure 2. Expression and secretion per cell were calculated by dividing densitometry by OD_600nm_ and normalizing to LB-L with no additives. Each data point is a replicate.

**Table S6.** Medium additives to evaluate the effect of phosphate buffer on expression and secretion titer. Species in each “level” were designed to have a similar buffer concentration and/or conductivity to the corresponding concentration of KH_2_PO_4_/K_2_HPO_4_.

| Level | [KH_2_PO_4_/K_2_HPO_4_] (mM) | [MOPS] (mM) | [NaCl] (mM) | Conductivity (mS/cm) |
| --- | --- | --- | --- | --- |
| 1 | 10 | 0 | 0 | 4 |
| 1 | 0 | 10 | 0 | 2.5 |
| 1 | 0 | 0 | 14 | 3.8 |
| 1 | 0 | 10 | 12 | 3.5 |
| 2 | 20 | 0 | 0 | 5.6 |
| 2 | 0 | 20 | 0 | 2.8 |
| 2 | 0 | 0 | 28 | 5.4 |
| 2 | 0 | 20 | 24 | 4.9 |
| 3 | 40 | 0 | 0 | 8.4 |
| 3 | 0 | 40 | 0 | 3.5 |
| 3 | 0 | 0 | 56 | 8.1 |
| 3 | 0 | 40 | 48 | 7.5 |
| 4 | 80 | 0 | 0 | 14 |
| 4 | 0 | 80 | 0 | 4.7 |
| 4 | 0 | 0 | 112 | 14 |
| 4 | 0 | 80 | 97 | 13 |
| 5 | 160 | 0 | 0 | 25 |
| 5 | 0 | 160 | 0 | 7.1 |
| 5 | 0 | 0 | 224 | 23 |
| 5 | 0 | 160 | 193 | 23 |
| LB-IM (6) | 0 | 0 | 291 | 31 |
| LB-L | 0 | 0 | 86 | 11 |


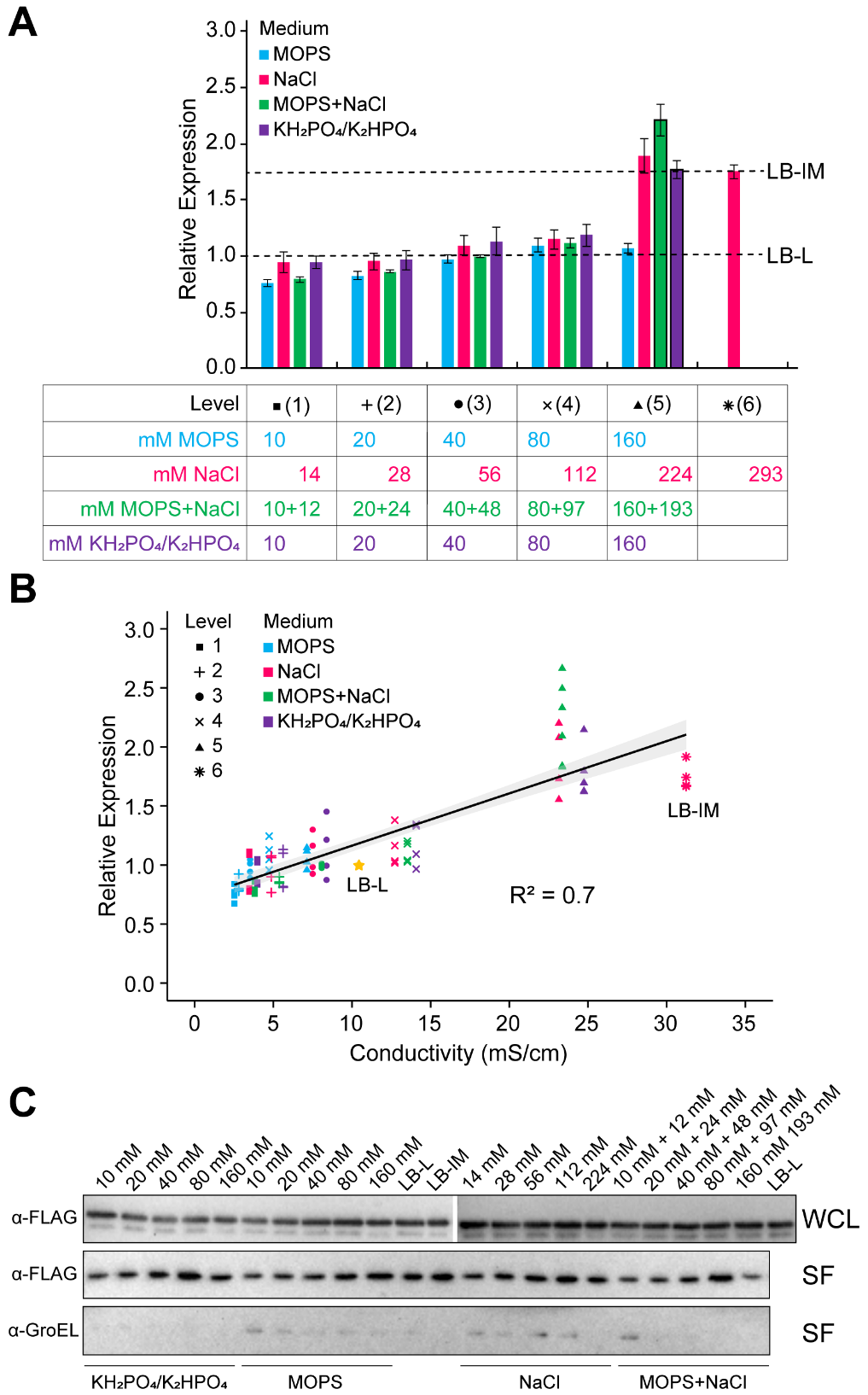


**Figure S5.** **A** Relative bulk expression of SptP-DH-2xFLAG in LB supplemented with the buffers and salts listed below the graph. Species in each “level” were designed to have a similar buffer concentration (MOPS, MOPS+NaCl) and/or conductivity (NaCl, MOPS+NaCl) to the corresponding concentration of KH_2_PO_4_/K_2_HPO_4_. Each column of the table corresponds to the group of bars above it, and each row of the column corresponds to a separate bar in the group. Data was normalized to LB-L using semi-quantitative western blotting. Error bars represent standard error for four biological replicates except for black-outlined bars, which represent six biological replicates. LB-L and LB-IM are denoted by dashed lines. **B** Relative bulk expression versus conductivity for the media in part **A.** The gray shading represents a 95% CI on the linear regression. Each data point is a replicate except for LB-L, which is labeled as a reference. **C** Western blots representative of four biological replicates. “WCL” is whole culture lysate and “SF” is secreted fraction.

**Table S7.** Conductivity of media at start of experiment and OD_600nm_ and secreted fraction pH at time of harvest for LB supplemented with buffers, salts, and carbon sources.

| Carbon Source | Medium | [MOPS]  (mM) | [NaCl]  (mM) | [KH_2_PO_4_/  K_2_HPO_4_]  (mM) | Conductivity  (mS/cm) | pH  at 8 hr | OD_600nm_  at 8 hr |
| --- | --- | --- | --- | --- | --- | --- | --- |
| None | MOPS | 90 | 0 | 0 | 5.0 | 7.58 ± 0.01 | 2.82 ± 0.04 |
|  | NaCl | 0 | 234 | 0 | 22 | 7.94 ± 0.07 | 2.33 ± 0.04 |
|  | MOPS+NaCl | 90 | 199 | 0 | 20 | 7.29 ± 0.01 | 2.77 ± 0.15 |
|  | KH_2_PO_4_/K_2_HPO_4_ | 0 | 0 | 90 | 14 | 7.51 ± 0.04 | 2.49 ± 0.05 |
| Glucose | MOPS | 90 | 0 | 0 | 5.4 | 6.74 ± 0.06 | 4.98 ± 0.04 |
|  | NaCl | 0 | 234 | 0 | 22 | 4.57 ± 0.02 | 3.06 ± 0.12 |
|  | MOPS+NaCl | 90 | 199 | 0 | 20 | 6.34 ± 0.04 | 4.51 ± 0.17 |
|  | KH_2_PO_4_/K_2_HPO_4_ | 0 | 0 | 90 | 14 | 6.50 ± 0.03 | 4.25 ± 0.22 |
| Glycerol | MOPS | 90 | 0 | 0 | 5.4 | 6.85 ± 0.02 | 3.42 ± 0.09 |
|  | NaCl | 0 | 234 | 0 | 21 | 4.82 ± 0.01 | 3.17 ± 0.28 |
|  | MOPS+NaCl | 90 | 199 | 0 | 20 | 6.78 ± 0.01 | 3.50 ± 0.29 |
|  | KH_2_PO_4_/K_2_HPO_4_ | 0 | 0 | 90 | 14 | 6.62 ± 0.01 | 3.13 ± 0.03 |


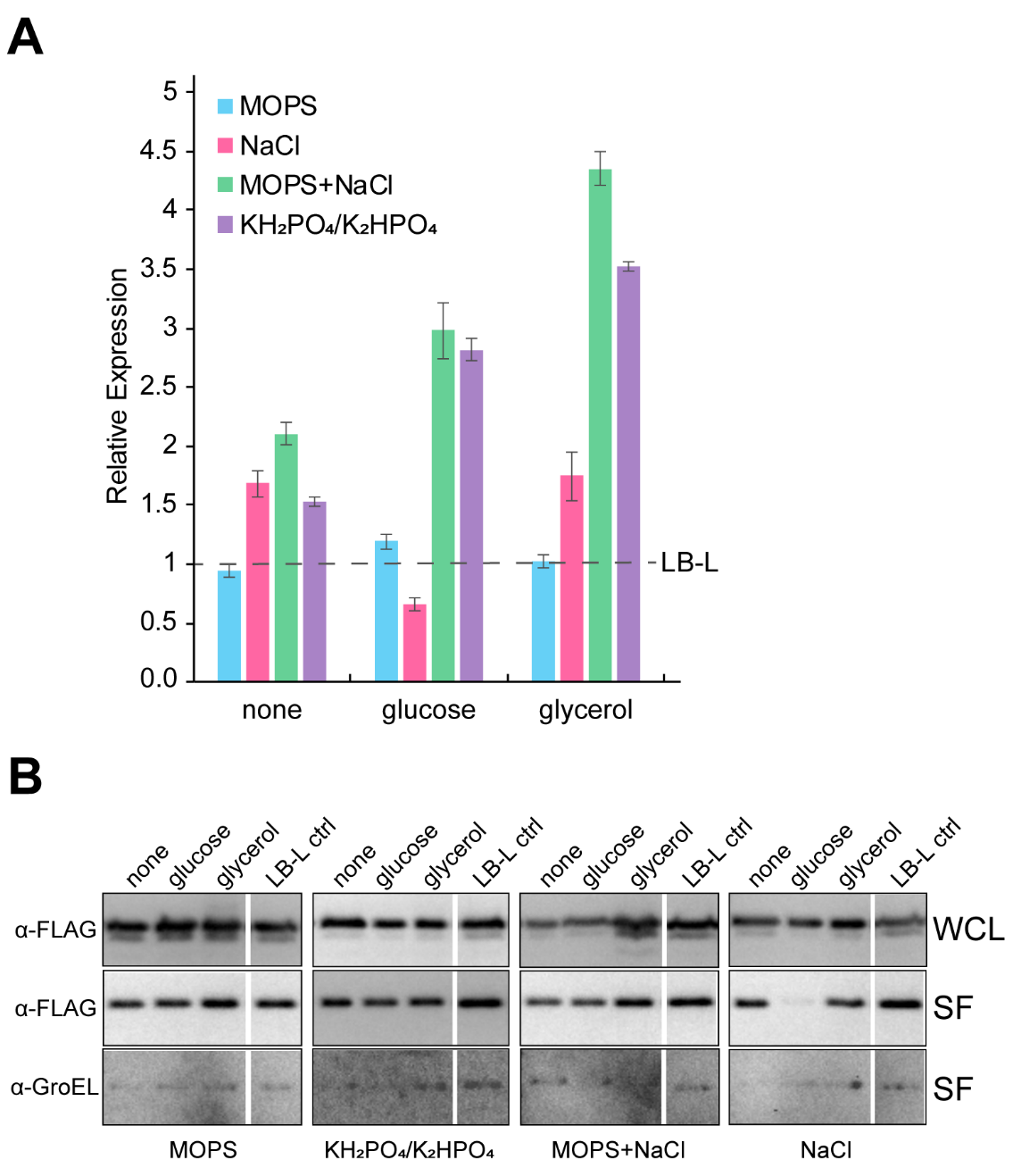


**Figure S6.** **A** Relative bulk expression of SptP-DH-2xFLAG-6xHis in media containing 10 g/L tryptone, 5 g/L yeast extract, and the additives listed in Supplementary Table 4. Bulk expression was normalized to LB-L with no additives (dotted line). Error bars represent standard error of the mean for three biological replicates. **B** Western blots are representative of three biological replicates. Samples were diluted to fall within the linear range of the LB-L signal. Boxed bands are from the same blot but were rearranged for clarity. “WCL” is whole culture lysate and “SF” is secreted fraction.


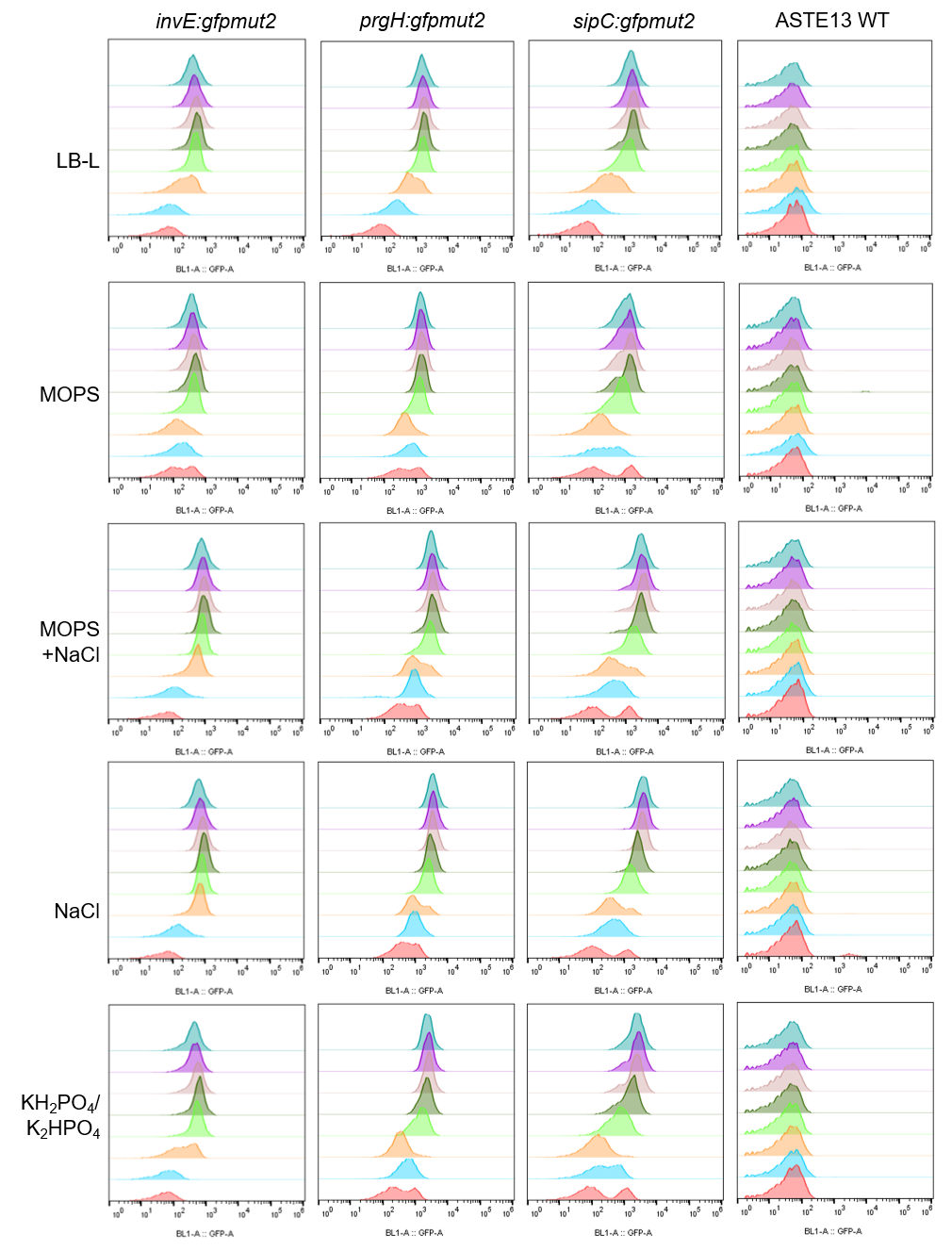


**Figure S7**. Representative histograms of GFP signal for flow cytometry to measure SPI-1 transcriptional activity in the media listed in Supplementary Table 4 with no added carbon source (“none”; Figure 5). Overlaid populations represent hourly time points from 1 hour (red) to 8 hours (teal). “ASTE13 WT” is a negative control.


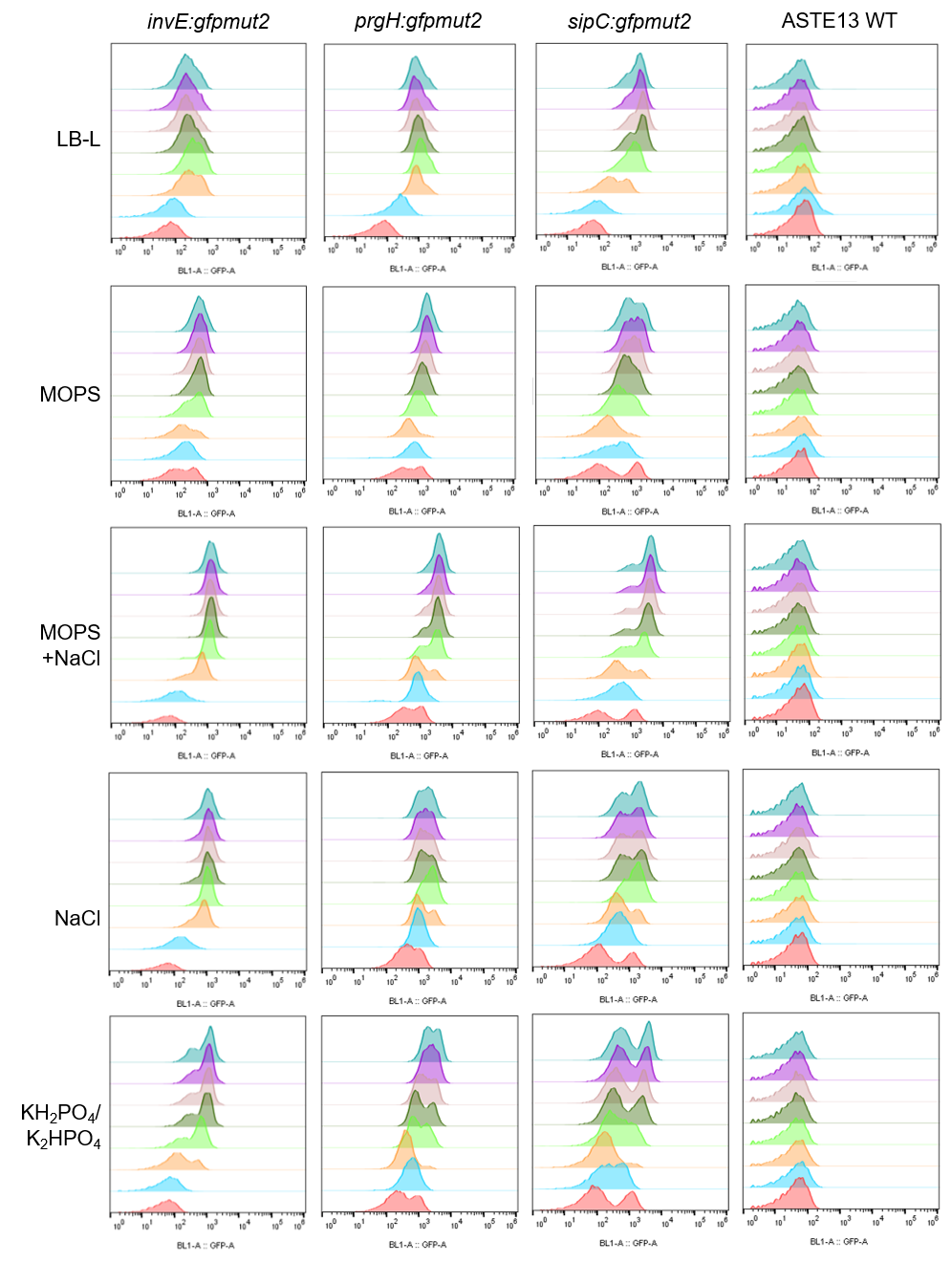


**Figure S8.** Representative histograms of GFP signal for flow cytometry to measure SPI-1 transcriptional activity in the media listed in Supplementary Table 4 with 0.4% w/v glucose (“glucose”; Figure 5). Overlaid populations represent hourly time points from 1 hour (red) to 8 hours (teal). “ASTE13 WT” is a negative control.


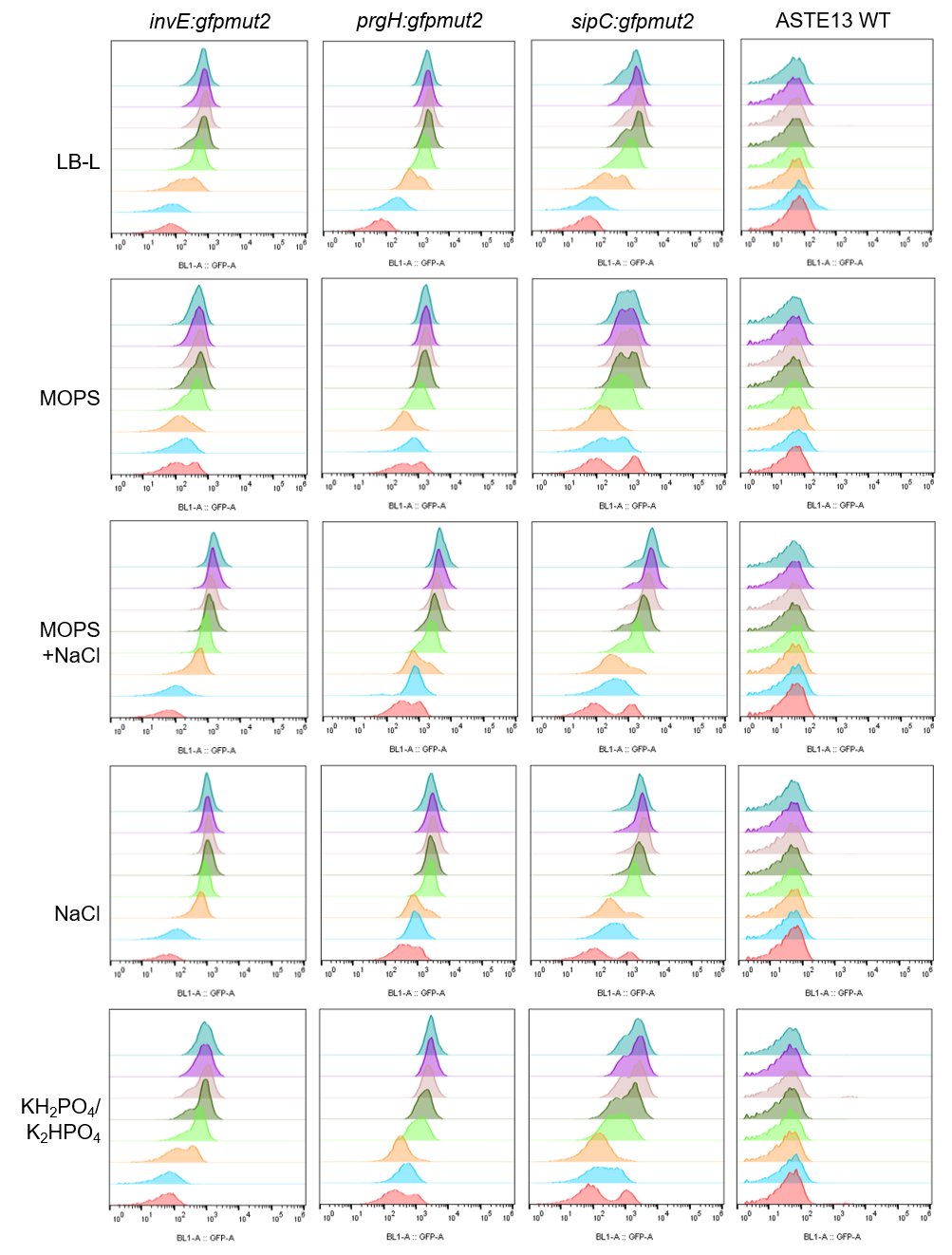


**Figure S9.** Representative histograms of GFP signal for flow cytometry to measure SPI-1 transcriptional activity in the media listed in Supplementary Table 4 with 0.4% w/v glycerol (“glycerol”; Figure 5). Overlaid populations represent hourly time points from 1 hour (red) to 8 hours (teal). “ASTE13 WT” is a negative control.


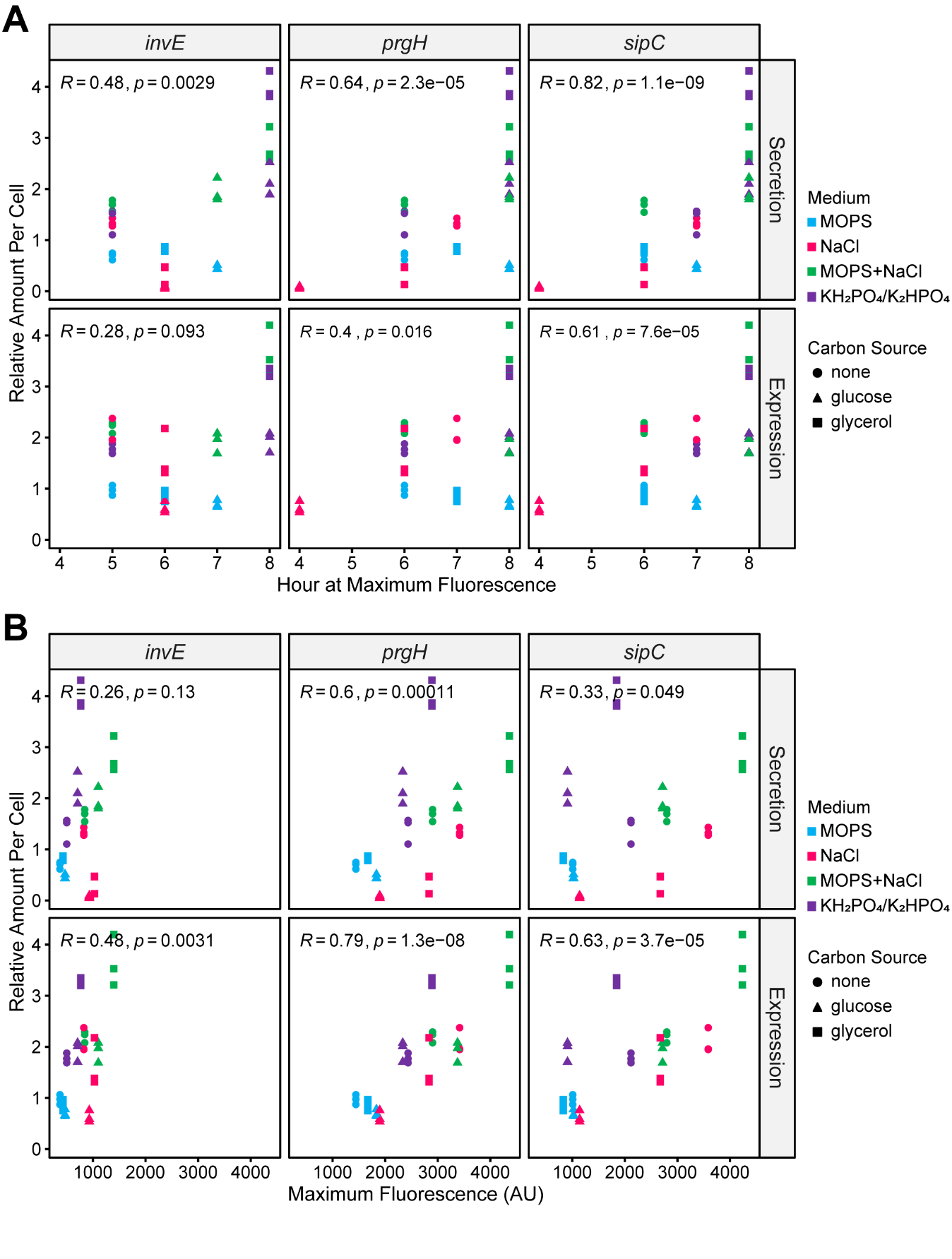


**Figure S10.** **A** Spearman correlations between expression or secretion per cell and hour at maximum mean fluorescence from flow cytometry in Figure 5. **B** Spearman correlations between expression or secretion per cell and maximum mean fluorescence from flow cytometry in Figure 5. Expression and secretion per cell were calculated by dividing densitometry by OD_600nm_ and normalizing to LB-L with no additives. Each data point is a replicate.


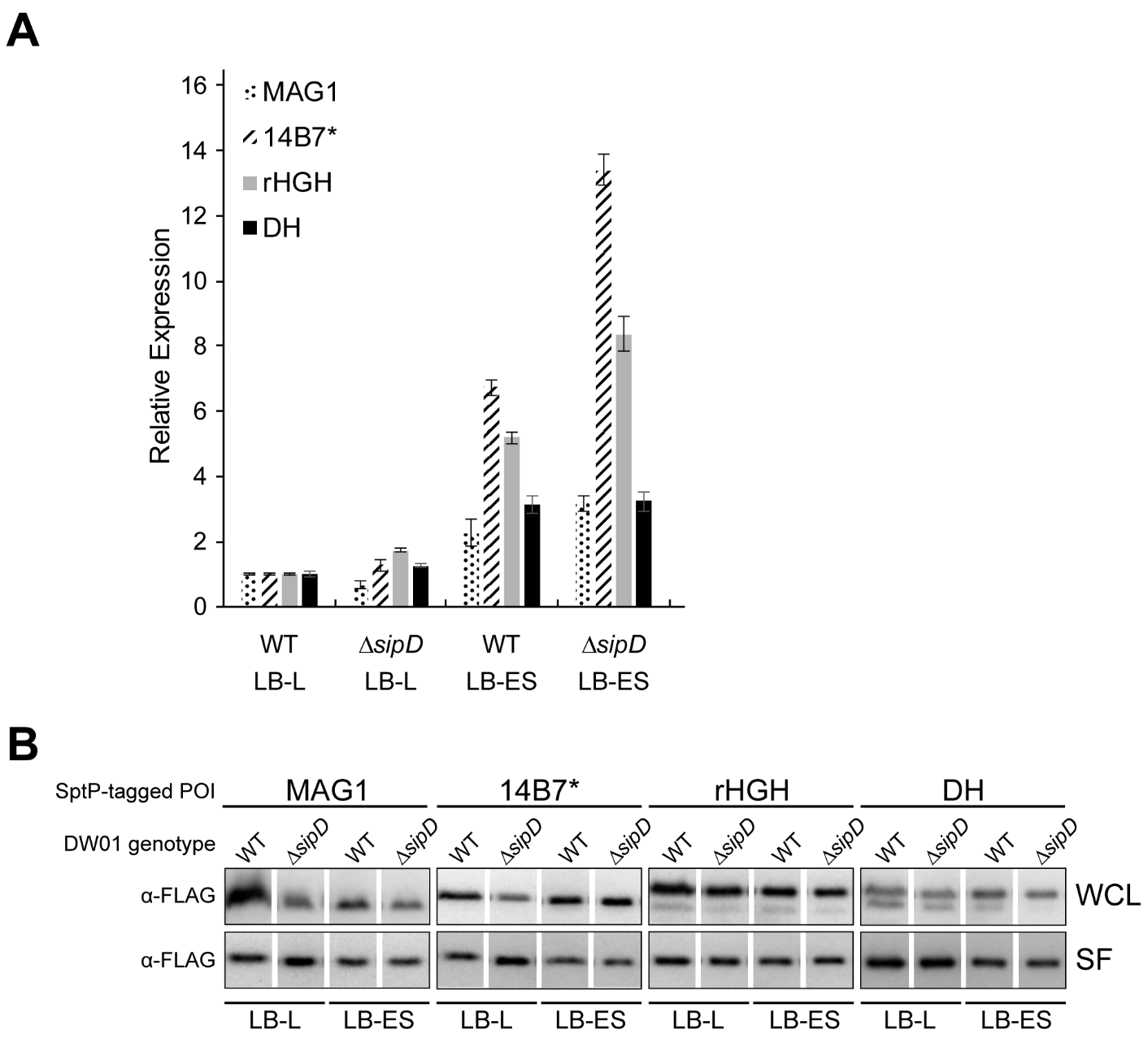


**Figure S11.** **A** Relative bulk expression of test proteins from WT and *∆sipD* strains in LB-L or LB-ES media. “LB-ES” is 10 g/L tryptone, 5 g/L yeast extract, 80 mM KH_2_PO_4_/K_2_HPO_4_ pH 7.4, 90 mM NaCl, and 0.4% w/v glycerol. Relative bulk expression was normalized to secretion from ASTE13 WT in LB-L for each protein using semi-quantitative western blotting. Error bars represent standard error of the mean for three biological replicates. **B** Western blots are representative of three biological replicates. Samples were diluted to fall within the linear range of the normalization signal. Boxed bands are from the same blot but were rearranged for clarity. “WCL” is whole culture lysate and “SF” is secreted fraction.


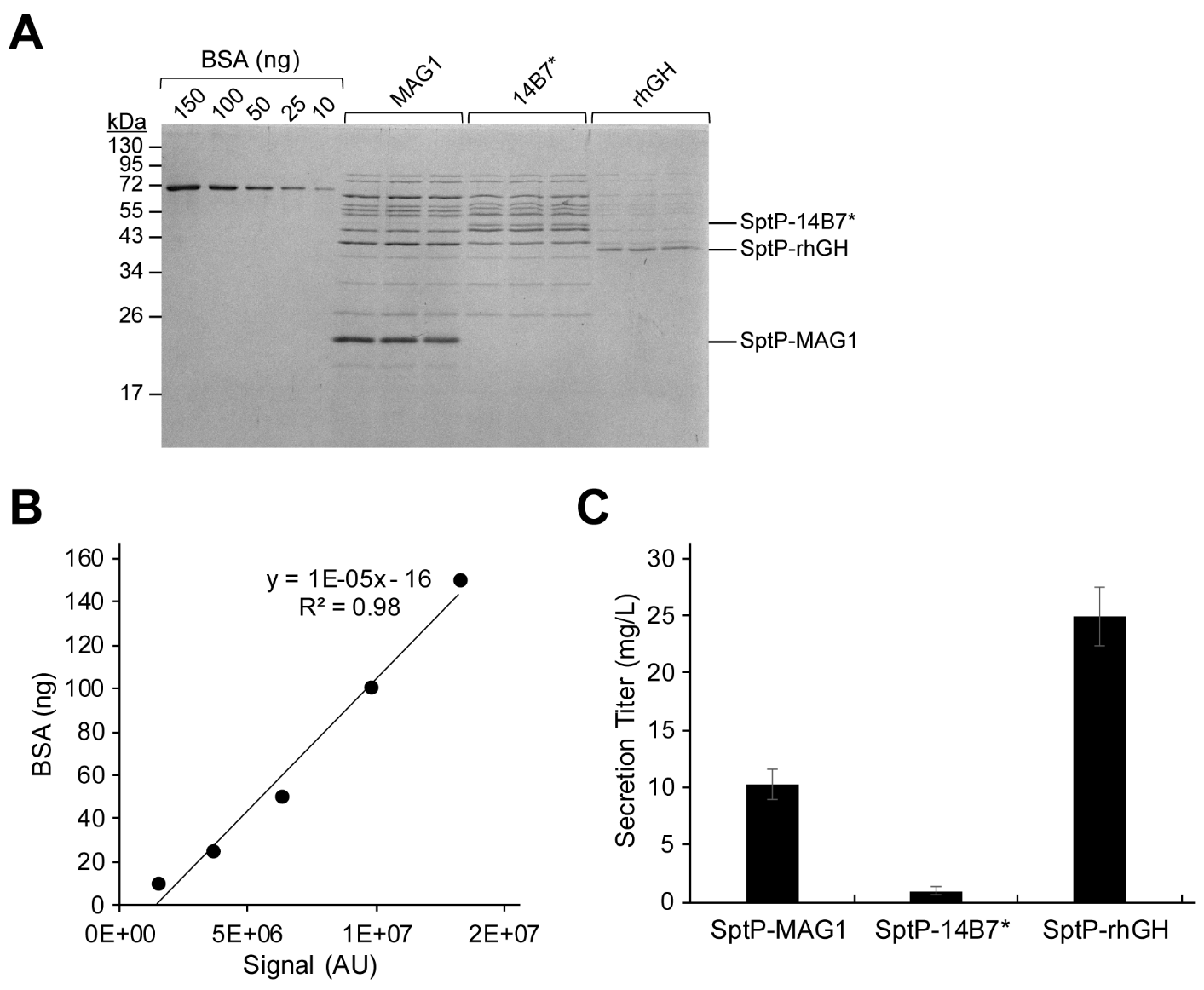
 **Figure S12 Titer of secreted proteins.** MAG1, 14B7*, and rhGH were secreted from an ASTE13 *∆sipD* strain in LB-ES. All proteins were in the format SptP-POI-2xFLAG-6xHis. Secretion titer (**C**) was measured by performing densitometry relative to a BSA standard curve (**B**) on a Coomassie-stained gel (**A**). The rhGH secreted fractions were diluted fivefold relative to those of MAG1 and 14B7*. Error bars represent one standard deviation.

**References**

1. Song M, Sukovich DJ, Ciccarelli L, Mayr J, Fernandez-Rodriguez J, Mirsky EA, et al. Control of type III protein secretion using a minimal genetic system. Nature Communications. 2017;8:14737.

2. Glasgow AA, Wong HT, Tullman-Ercek D. A Secretion-Amplification Role for Salmonella enterica Translocon Protein SipD. ACS Synth Biol. 2017;6:1006–15.

3. Metcalf KJ, Finnerty C, Azam A, Valdivia E, Tullman-Ercek D. Using Transcriptional Control To Increase Titers of Secreted Heterologous Proteins by the Type III Secretion System. Applied and Environmental Microbiology. 2014;80:5927–34.

4. Metcalf KJ, Bevington JL, Rosales SL, Burdette LA, Valdivia E, Tullman-Ercek D. Proteins adopt functionally active conformations after type III secretion. Microbial Cell Factories. 2016;15:213.
